# Accurate external localization of the left frontal cortex in dogs by using pointer based frameless neuronavigation

**DOI:** 10.7717/peerj.3425

**Published:** 2017-07-10

**Authors:** Robrecht Dockx, Kathelijne Peremans, Romain Duprat, Lise Vlerick, Nick Van Laeken, Jimmy H. Saunders, Ingeborgh Polis, Filip De Vos, Chris Baeken

**Affiliations:** 1 Department of Psychiatry and Medical Psychology, Ghent University, Ghent, East-Flanders, Belgium; 2 Faculty of Veterinary Medicine, Ghent University, Merelbeke, East-Flanders, Belgium; 3 Faculty of Pharmaceutical Sciences, Ghent University, Ghent, East-Flanders, Belgium

**Keywords:** Canine, Non-stereotactic, Brain, Neuronavigation, TMS, Neuropsychiatric disorders

## Abstract

**Background:**

In humans, non-stereotactic frameless neuronavigation systems are used as a topographical tool for non-invasive brain stimulation methods such as Transcranial Magnetic Stimulation (TMS). TMS studies in dogs may provide treatment modalities for several neuropsychological disorders in dogs. Nevertheless, an accurate non-invasive localization of a stimulation target has not yet been performed in this species.

**Hypothesis:**

This study was primarily put forward to externally locate the left frontal cortex in 18 healthy dogs by means of a human non-stereotactic neuronavigation system. Secondly, the accuracy of the external localization was assessed.

**Animals:**

A total of 18 healthy dogs, drawn at random from the research colony present at the faculty of Veterinary Medicine (Ghent University), were used.

**Methods:**

Two sets of coordinates (*X*, *Y*, *Z* and *X*″, *Y*″, *Z*″) were compared on each dog their tomographical dataset.

**Results:**

The non-stereotactic neuronavigation system was able to externally locate the frontal cortex in dogs with accuracy comparable with human studies.

**Conclusion and clinical importance:**

This result indicates that a non-stereotactic neuronavigation system can accurately externally locate the left frontal cortex and paves the way to use guided non-invasive brain stimulation methods as an alternative treatment procedure for neurological and behavioral disorders in dogs. This technique could, in analogy with human guided non-invasive brain stimulation, provide a better treatment outcome for dogs suffering from anxiety disorders when compared to its non-guided alternative.

## Introduction

Neuronavigation systems provide a three dimensional orientation of the neurological structures contained in the skull and vertebral column. They can either be frame or frameless based and have currently their main application in canine neurosurgery. In this field, frame based neuronavigation uses a mechanical frame, mounted on the skull. This allows navigation along the dorsal, transversal and sagittal plane of the brain (Wininger, 2014). Frameless neuronavigation on the other hand does not require a mounted frame to locate a brain region. It uses external fiducial markers (natural occurring or artificially added) and infrared cameras. Both commercially available neuronavigation systems, including a veterinary-specific frameless neuronavigation system (Brainsight stereotactic vet system; Rogue Research, Montreal, Quebec), have been used for stereotactic neurologic procedures such as biopsy or injections in the canine brain (Chen et al., 2012; Flegel et al., 2003; Giroux et al., 2002; Koblik et al., 1999; Taylor et al., 2013; Troxel & Vite, 2008).

Neuronavigation is, besides in neurosurgery, used in human neuropsychiatry as a tool for non-invasive brain stimulation methods such as Transcranial Magnetic Stimulation (TMS) (Herwig et al., 2001). TMS has become a major player in the treatment modalities of neuropsychiatric conditions like depression and anxiety (Lefaucheur et al., 2014). In order to accurately and repeatedly stimulate the targeted neocortical regions, coil navigation methods such as MRI based—non-stereotactic frameless neuronavigation systems are essential (Klein et al., 2015). Moreover, a good localization of the target region provides a better treatment outcome (Schonfeldt-Lecuona et al., 2010).

In dogs with behavioral disorders, a deficiency in both neuronal function as well as the serotonergic system have been reported in the frontocortical region in general and the left in particular (Peremans et al., 2003; Vermeire et al., 2012; Vermeire et al., 2009a; Vermeire et al., 2009b). Guided non-invasive brain stimulation methods may therefore have a place in future treatments of drug resistant neuropsychiatric disorders in the canine species necessitating, similar to men, an accurate localization of the target region. A more accurate localization of the target region might, in analogy with humans, provide a better treatment outcome (Schonfeldt-Lecuona et al., 2010).

It is clear that feasibility and accuracy studies should precede guided non-invasive brain stimulation methods in dogs. However, to date, no studies have been conducted using a human non-stereotactic frameless neuronavigation system in dogs for non-invasive procedures such as TMS. Hence, the main purpose of this study was to test the feasibility of this type of neuronavigation system to externally locate the left frontal cortex. Additionally the accuracy of the external localization was determined by means of comparing the coordinates of the set target and its external location.

## Material and Method

### Animals

Eighteen dogs (two fox-hounds and 16 beagles; nine neutered males; eight neutered females and one intact female: aged between eight months and eight years old) were included in this study. The dogs were owned by the Ghent University department of Small Animal and the Ghent University department of Veterinary medical imaging and small animal orthopaedics. The Ghent University Ethical Committee approved this study and all guidelines for animal welfare, imposed by the Ethical Committee, were respected (EC 2015_38).

**Figure 1:**
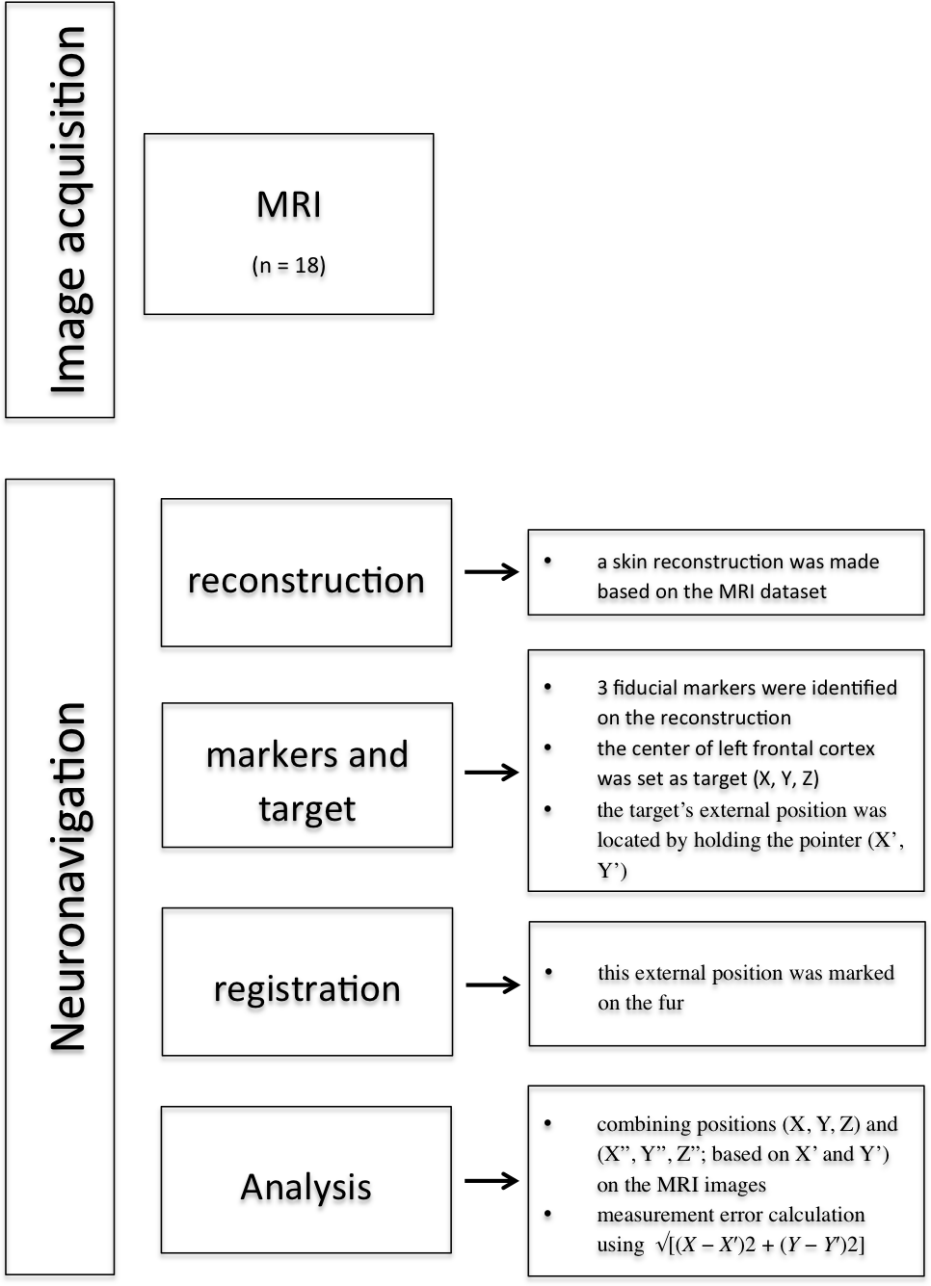
Overview methodology of the study.

**Figure 2:**
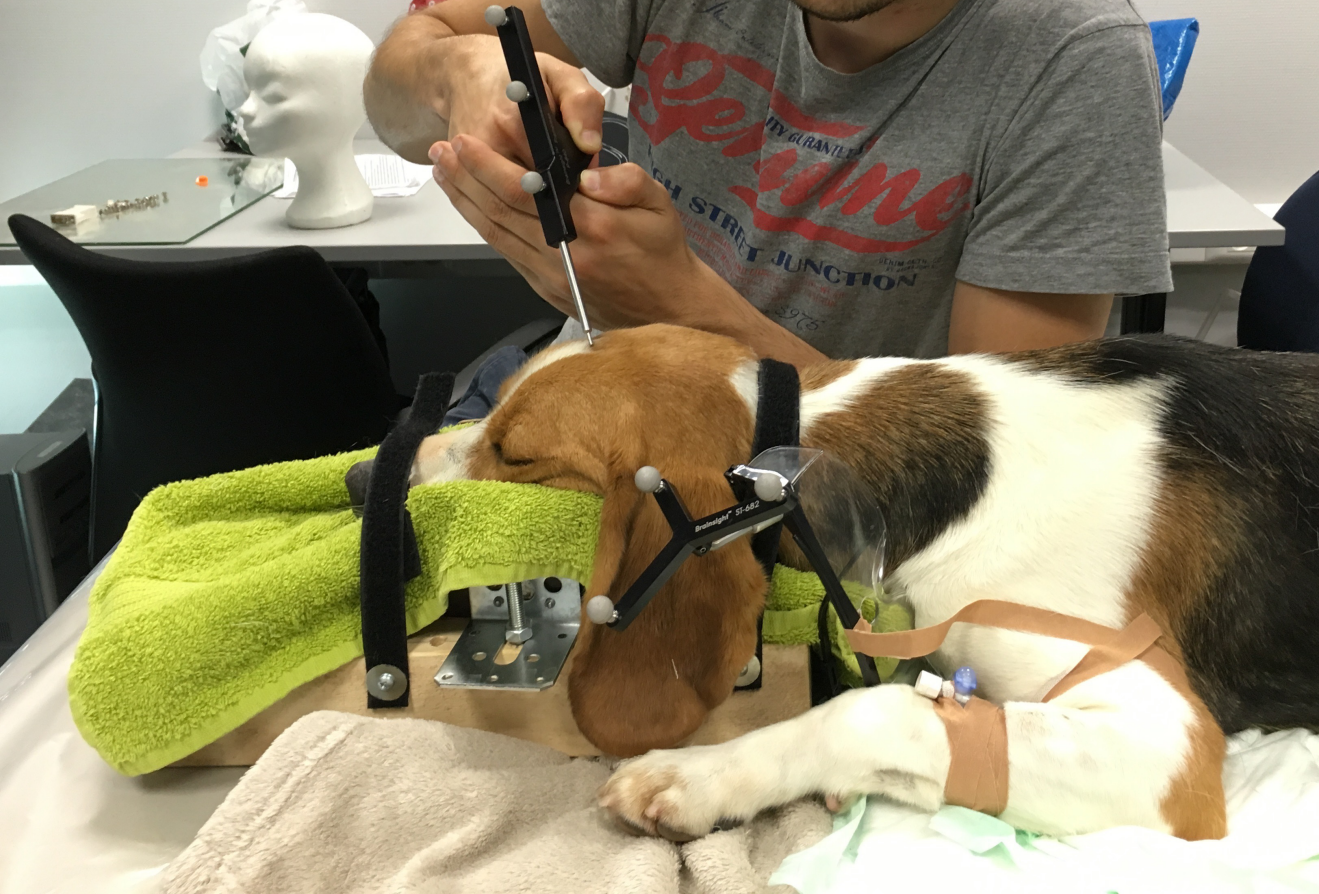
A beagle placed in sternal recumbence, with the head fixed in a self-made mould. The subject tracker is attached to the mould and the dog in the neck region. The infrared camera was placed in front of the dog.

### Neuronavigation protocol (Fig. 1)

In order to obtain a tomographical dataset, all dogs underwent magnetic resonance imaging (MRI). A Siemens 3T Magnetom Trio Tim system (Siemens Medical Systems, Erlangen, Germany), using the phased-array spine coil and a phased-array body matrix coil, was used to collect the data set. Following the placement of an intravenous cephalic catheter, the dogs were pre-medicated intramuscularly (IM) with dexmedetomidine (375 µg/m^2^ body surface, Dexdomitor^®^; Orion Corporation, Espoo, Finland). Propofol (Propovet Multidose^®^; Abbott Laboratories, Berkshire, UK, 1–2 mg/kg given to effect) was administered IV to induce general anaesthesia. Anaesthesia was maintained with isoflurane (Isoflo^®^; Abbott Laboratories, Berkshire, UK) in oxygen using a rebreathing system. The dogs were placed head first and sternally in the scanner bore. After completion of the MRI acquisition, the dogs were allowed to recover.

During this recovery phase, the dogs were placed in sternal recumbence, with the head positioned in a self-made mould (Fig. 2). The subject tracker was attached to the neck region in such a way that the infrared camera could detect the marker balls. If needed, the dogs were given dexmedetomidine (0,5–1 µg/kg; IV) to complete the following neuronavigation protocol.

First, the tomographical data were loaded into the neuronavigation system (Brainsight; Rogue-resolutions Ltd, Cardiff, UK). Using the software, a skin reconstruction was made. Next, three to four fiducial markers (landmarks) were created and identified on the skin reconstructions: the top of the nose, the external occipital protuberance and one or both medial corners of the eyes. In some dogs, the MRI coil caused a moderate to severe ventro-abaxial displacement of the skin, which hindered the identification of one of the medial corners of the eyes. In these cases the medial corner(s) were identified based on the MRI images instead of the skin reconstructions. Subsequently, the center of the left frontal cortex was set as target and was manually identified on the tomographical dataset. The center of the left frontal cortex was determined as the cortical region situated in the upper fourth part between the prorean sulcus and the rostral part of the lateral rhinal sulcus; halfway the distance from the caudal rim of the olfactory lobe and the presylvian sulcus. The target’s external position was located by holding a pointer—connected to three reflecting balls—perpendicular over the target region as indicated by the neuronavigation software. This external position was marked on the fur using a permanent marker. The position of the mark was then confirmed by once again locating the target’s external position with the pointer. At last, the position of the mark was registered in a Cartesian coordinate system. The *Y*-axis ran from the external occipital protuberance to the top of the nose, whereas the *X*-axis was placed perpendicular to the *Y*-axis containing the center of the externally placed mark.

### Accuracy assessment

**Figure 3:**
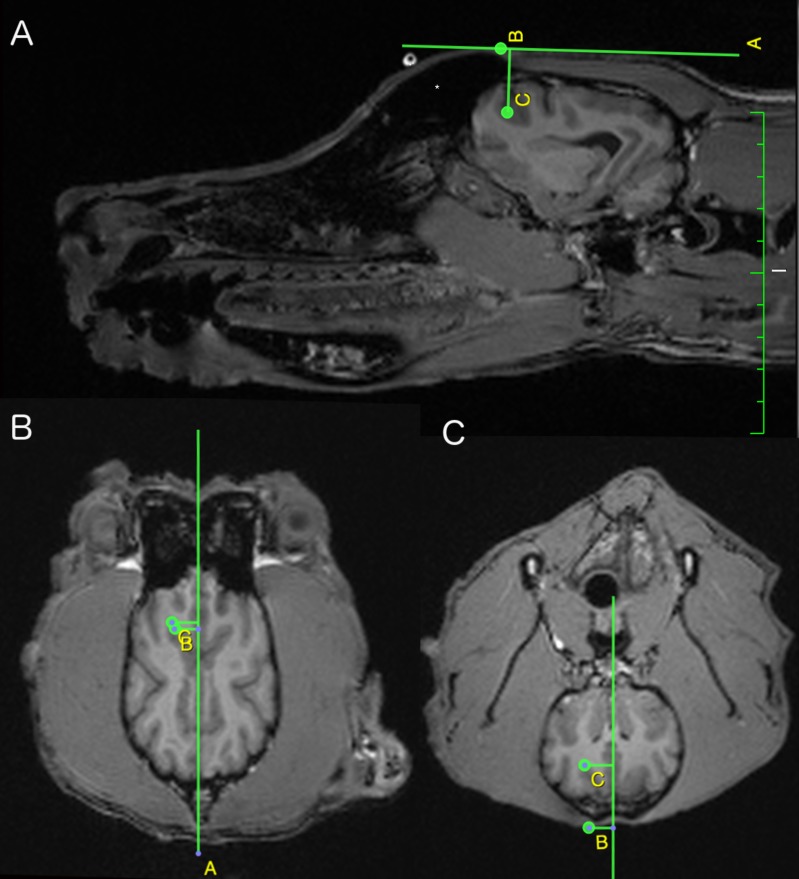
Sagittal (A), dorsal (B) and transversal (C) view of the canine brain at the level of the left frontal cortex. On all views, the yellow letters B and C indicate the target’s external position on the skull and the center of the left frontal cortex (set as target for the neuronavigation) respectively. The sagittal view indicates the depth (C-B (yellow lettering)) and the rostro-caudal deviation (C minus B (yellow lettering). The dorsal view shows the latero-lateral deviation (C minus B (yellow lettering)) and again the rostro-caudal deviation (B-C (yellow lettering)). The transversal view provides the depth (B-C (yellow lettering)) and again the latero-lateral deviation (C minus B (yellow lettering)). (*Left part of the frontal sinus).

In this cross-sectional study, the accuracy of a human frameless neuronavigation system was assessed by comparing two sets of coordinates. The first set of coordinates (*X*, *Y*, *Z*) was obtained by identifying the center of the target region on the tomographical dataset. The Cartesian coordinate system, used to measure the target’s external position, provided the second set (*X*′, *Y*′). This second set was plotted onto the tomographical dataset, creating a third set of coordinates (*X*″, *Y*″, *Z*″). The average difference, measured in centimeters, between the two sets of coordinates was calculated for each dog on the dorsal plane of the skull (Fig. 3). This provided the latero-lateral deviation (*X* − *X*″) and the rostro-caudal deviation (*Y* − *Y*″). The measurement error was calculated for each dog using the Pythagorean theorem $\sqrt{}[(X-{X}^{{^{\prime}}})^{2}+(Y-{Y}^{{^{\prime}}})^{2}]$. The dorso-ventral deviation (*Z* − *Z*″) was not incorporated in this theorem. It was hypothesized that the median measurement error could not differ from zero. Osirix 6.5.2 was used to calculate the differences between the two sets of coordinates.

### Stimulation depth

In addition the depth (dorso-ventral distance) was assessed to evaluate the feasibility of TMS in dogs. Osirix 6.5.2 was used to calculate the depth between the two sets of coordinates.

### Statistical analysis

SPSS 23 (SPSS Inc, Chicago, IL, USA) was used to compute all the performed tests. Normality was tested by executing the Shapiro–Wilk test (significance set at *p* < 0.05). Outliers were identified and removed from the dataset. To assess the measurement error, a Wilcoxon one-sample signed rank test was used with a hypothesized median of 0.00 cm. In addition, a Wilcoxon signed rank test was put forward to check for differences between each rostro-caudal distance, latero-lateral distance and depth measured on its two different planes.

## Results

The Shapiro–Wilk test revealed data that were not normally distributed. Dog number 2, 7 and 12 were identified as outliers and pairwise removed from the dataset. The Wilcoxon one-sample signed rank test showed a statistically significant difference (*p* < 0.001) between the hypothesized median measurement error (0.00 cm) and the actual measurement error between the left frontal cortex and its external location on the skull. The actual median measurement error was 0.26 cm. The average measurement error constrains 0.36 cm (sd = 0.22 cm, ranged from 0.14 cm to 0.712 cm, 95% CI [−0.06; 0.79]).

Table 1 shows the mean differences between the two sets of coordinates on each view. No significant differences were found between each rostro-caudal distance, latero-lateral distance and depth measured on its two different planes.

**Table 1:** The mean differences (in cm) between the two sets of coordinates for each dog on the dorsal, sagittal and transversal plane of the skull.

Plane	Distance	Mean	SD	SE	Min	Max
DORSAL	LATERO–LATERAL	0.17	0.13	0.03	0.05	0.53
	ROSTRO–CAUDAL	0.31	0.21	0.05	0.08	0.68
SAGITTAL	DEPTH	1.76	0.40	0.09	1.30	3,1
	ROSTRO–CAUDAL	0.35	0.18	0.05	0.11	0.66
TRANSVERSAL	DEPTH	1.79	0.35	0.08	1.33	2.90
	LATERO–LATERAL	0.16	0.15	0.04	0.03	0.55

Between the left frontal cortex and its external location on the skull there is an average deviation in latero-lateral distance of 0.17, sd = 0.14 cm. The rostro-caudal difference between both is 0.33, sd = 0.19 cm. The stimulation depth was on average 1.78 cm, sd 0.37 cm.

## Discussion

An external location was found for the set target in each dog by means of the used neuronavigation method, which could allow non-invasive brain stimulation methods such as guided rTMS. These techniques could therefore be used in future treatments of neuropsychiatric disorders in dogs.

Three dogs were identified in the dataset as outliers. Movements of the dog or disturbance of the digital reference frame were most likely to create these outliers. It was hypothesized that the median measurement error would not differ significantly from zero (hypothesized median measurement error).

In contrast to what was expected, the median measurement error differed significantly from zero. Hereby, it could be concluded that this type of neuronavigation system is not accurate in dogs and thereby not feasible to use in this species. However, a measurement error of less than 0.3 cm is accepted for the use of a neuronavigation system to obtain intracranial biopsies (Bjartmarz & Rehncrona, 2007; Dorward et al., 1999; Gumprecht, Widenka & Lumenta, 1999; Machado & Zrinzo, 2013; Spetzger et al., 2002; Woerdeman et al., 2007; Zinreich et al., 1993). In analogue, this study was able to localize the left frontal cortex external position in dogs with a median measurement error of 0.26 cm (average 0.36 cm). Thus, it appears that this study was able to determine the left frontal cortex’s external position in dogs. In comparison, the Brainsight stereotactic vet system was able to place Deep Brain Stimulation (DBS) electrodes with an accuracy of 0.46 cm (sd = 0.15 cm) in normal dogs (Long et al., 2014) and contains an upper bound needle placement error limit of 0.331 cm (Chen et al., 2012). This implies that a human frameless non-stereotactic neuronavigation system can be as accurate as a neuronavigation system specifically developed for stereotactic veterinary use. However, the dog’s frontal lobe is considerably smaller in volume when compared to its human counterpart (Tapp et al., 2004). This may require a higher level of accuracy when externally locating the frontal cortex in dogs.

On average, the depth of the center of the frontal cortex in dogs constrains 1.78 cm (range from 1.30 cm to 3.10 cm). In comparison, the center of the frontal cortex in humans has a depth ranging from 2.00 cm to 3.00 cm. Despite the obvious discrepancy between a human and beagle head, a comparable target depth for the frontal cortex is present. This similar average target depth could be explained by the presence of a large frontal sinus in dogs, as depicted in Fig. 3. The frontal sinus could comprise a distinguished amount of the distance between the scalp and the center of the target, provoking an increase in scalp-cortex distance. In its turn, an enlargement of the scalp-cortex region can elicit differences in the magnetic field strength created—during a TMS protocol—in the underlying cortical region. Furthermore, at depths up to 1.5 cm, the magnetic field strength—created by classic TMS figure-of-eight coil at an intensity of 120% motor threshold—remains above the threshold of neuronal stimulation (Roth et al., 2007). This implies that the presence of a large frontal sinus might limit the possibility to transcranially stimulate distant brain regions.

When assessing the measurement error, this study only included the latero-lateral (*X* − *X*″) deviation and the rostro-caudal (*Y* − *Y*″) deviation. The dorso-ventral (*Z* − *Z*″) deviation was not included. This was because the scope of this study was to superficially determine the external localization of the frontal cortex. Adding the dorso-ventral deviation to the theorem would greatly negatively influence the outcome. When performing accuracy studies of frameless neuronavigation systems, it must be kept in mind that its accuracy is influenced by user dependent (measuring errors, handler) and mechanical errors (technical limitations and imaging procedure), which makes it implausible to achieve the predefined hypothetical difference of zero (Sparing et al., 2008; Spetzger et al., 2002; Steinmeier et al., 2000). A second limitation was created while obtaining the tomographical dataset. The used coils caused, during the MRI acquisition, a moderate to severe ventro-abaxial displacement of the skin. This displacement was noticeable on the skin reconstruction, which hindered the identification of the fiducial markers. In addition, the chosen fiducial markers were closely located to one other, which might negatively influence the accuracy of the external localization (Steinmeier et al., 2000). On the other hand, artificial fiducial markers, which should be attached to the head, could negatively influence the accuracy of the neuronavigation system (Chen et al., 2012).

To conclude, this study was able to externally locate the left frontal cortex in dogs using a human commercially available neuronavigation system with a high level of accuracy. This could allow non-invasive brain stimulation methods such as guided rTMS to be used in future treatments of neuropsychiatric disorders in dogs. Error calculations, to eliminate the machine error, combined with artificial fiducial markers could augment the found accuracy of non-stereotactic frameless neuronavigation in dogs.

##  Supplemental Information

10.7717/peerj.3425/supp-1 Data S1 Raw data analysis

10.7717/peerj.3425/supp-2 Data S2 Raw data

## List of abbreviations

TMS: Transcranial Magnetic Stimulation
MRI: Magnetic Resonance Imaging

## Additional Information and Declarations

### Data Availability

The following information was supplied regarding data availability: The raw data has been supplied as a  Supplementary File.
