# Supplementary material for: Accurate external localization of the left frontal cortex in dogs by using pointer based frameless neuronavigation
Source: PeerJ. 2017 Jul 10;5:e3425. doi: 10.7717/peerj.3425 (PMC5507169; doi:10.7717/peerj.3425)
Supplement: Data S1 [file peerj-05-3425-s001.pdf]

```
DATASET ACTIVATE DataSet3.
DATASET CLOSE DataSet4.
GET DATA /TYPE=XLSX
  /FILE='/Volumes/External hard drive/raw_data_neuronavigation.xlsx'
  /SHEET=name 'sheet 2'
  /CELLRANGE=full
  /READNAMES=on
  /ASSUMEDSTRWIDTH=32767.
EXECUTE.
DATASET NAME DataSet6 WINDOW=FRONT.
DATASET ACTIVATE DataSet3.
DATASET CLOSE DataSet6.
GET DATA /TYPE=XLSX
  /FILE='/Volumes/External hard drive/raw_data_neuronavigation.xlsx'
  /SHEET=name 'sheet 2'
  /CELLRANGE=full
  /READNAMES=on
  /ASSUMEDSTRWIDTH=32767.
EXECUTE.
DATASET NAME DataSet7 WINDOW=FRONT.
DATASET ACTIVATE DataSet3.
EXAMINE VARIABLES=sqrtXenY
  /PLOT BOXPLOT HISTOGRAM NPPLOT
  /COMPARE GROUPS
  /STATISTICS DESCRIPTIVES
  /CINTERVAL 95
  /MISSING LISTWISE
  /NOTOTAL.
```

Explore

Notes

Output Created		10-APR-2017 13:55:...
Comments		
Input	Active Dataset	DataSet3
	Filter	sqrtXenY < 0.94 (FILTER)
	Weight	<none>
	Split File	<none>
	N of Rows in Working Data File	15
Missing Value Handling	Definition of Missing	User-defined missing values for dependent variables are treated as missing.
	Cases Used	Statistics are based on cases with no missing values for any dependent variable or factor used.
Syntax		EXAMINE VARIABLES=sqrtXenY /PLOT BOXPLOT HISTOGRAM NPPLOT /COMPARE GROUPS /STATISTICS DESCRIPTIVES /CINTERVAL 95 /MISSING LISTWISE /NOTOTAL.
Resources	Processor Time	00:00:00.64
	Elapsed Time	00:00:01.00

Case Processing Summary

	Cases					
	Valid		Missing		Total	
	N	Percent	N	Percent	N	Percent
sqrt (X en Y)	15	100.0%	0	0.0%	15	100.0%

Descriptives

			Statistic	Std. Error
sqrt (X en Y)	Mean		.3642	.05565
	95% Confidence Interval for Mean	Lower Bound	.2449	
		Upper Bound	.4836	
	5% Trimmed Mean		.3576	
	Median		.2584	
	Variance		.046	
	Std. Deviation		.21554	
	Minimum		.13	
	Maximum		.71	
	Range		.58	
	Interquartile Range		.44	
	Skewness		.747	.580
	Kurtosis		-1.368	1.121

Tests of Normality

	Kolmogorov-Smirnov[a]			Shapiro-Wilk		
	Statistic	df	Sig.	Statistic	df	Sig.
sqrt (X en Y)	.307	15	.000	.793	15	.003

a. Lilliefors Significance Correction

sqrt (X en Y)

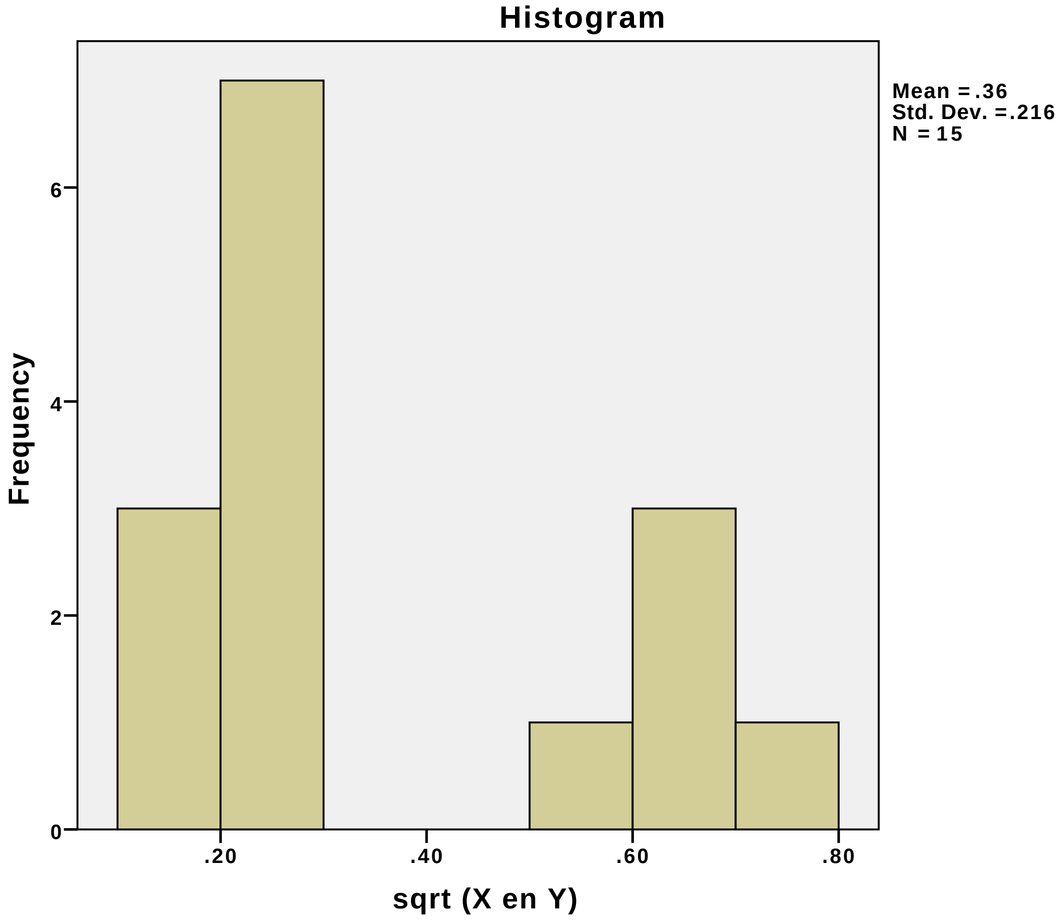
Histogram
Mean = .36
Std. Dev. = .216
N = 15
Frequency
0
2
4
6
.20
.40
.60
.80
sqrt (X en Y)

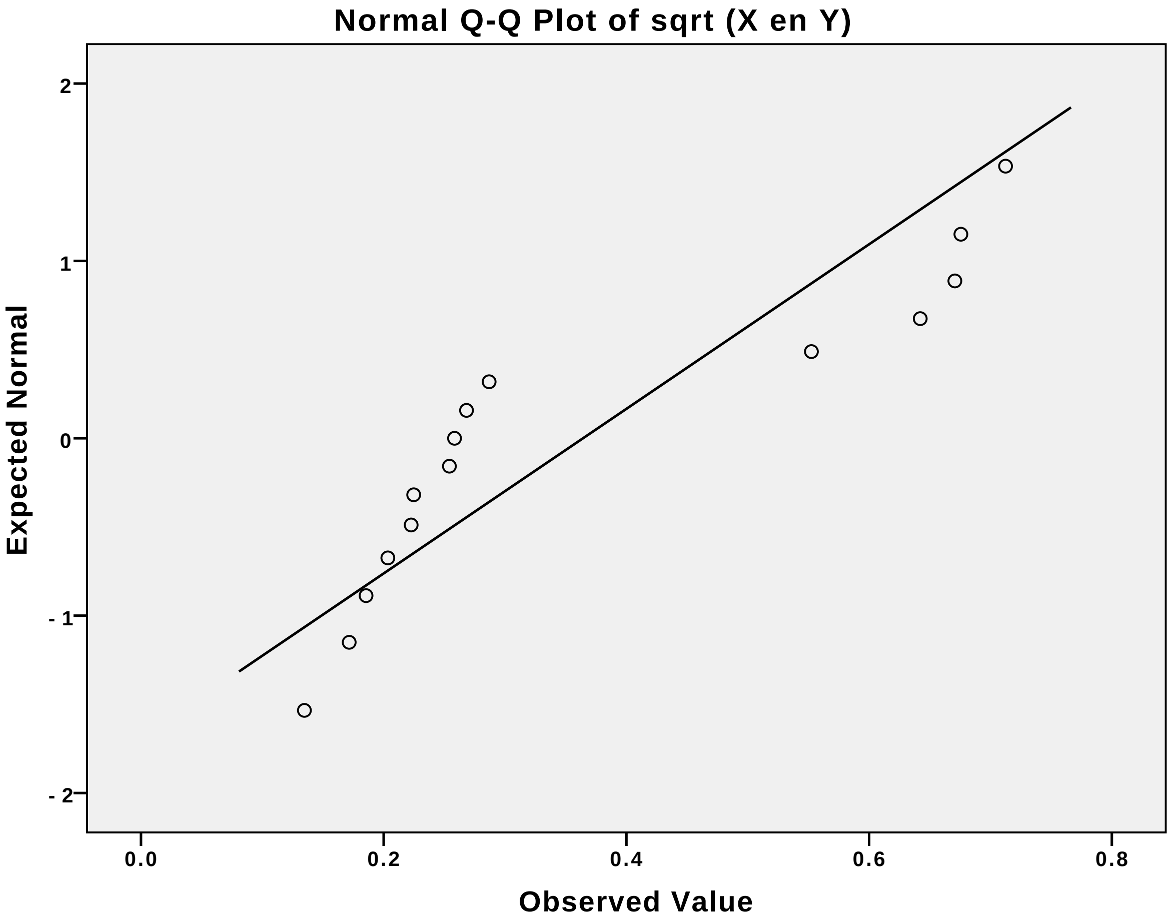
Normal Q-Q Plot of sqrt (X en Y)
2
1
0
- 1
- 2
Expected Normal
0.0
0.2
0.4
0.6
0.8
Observed Value

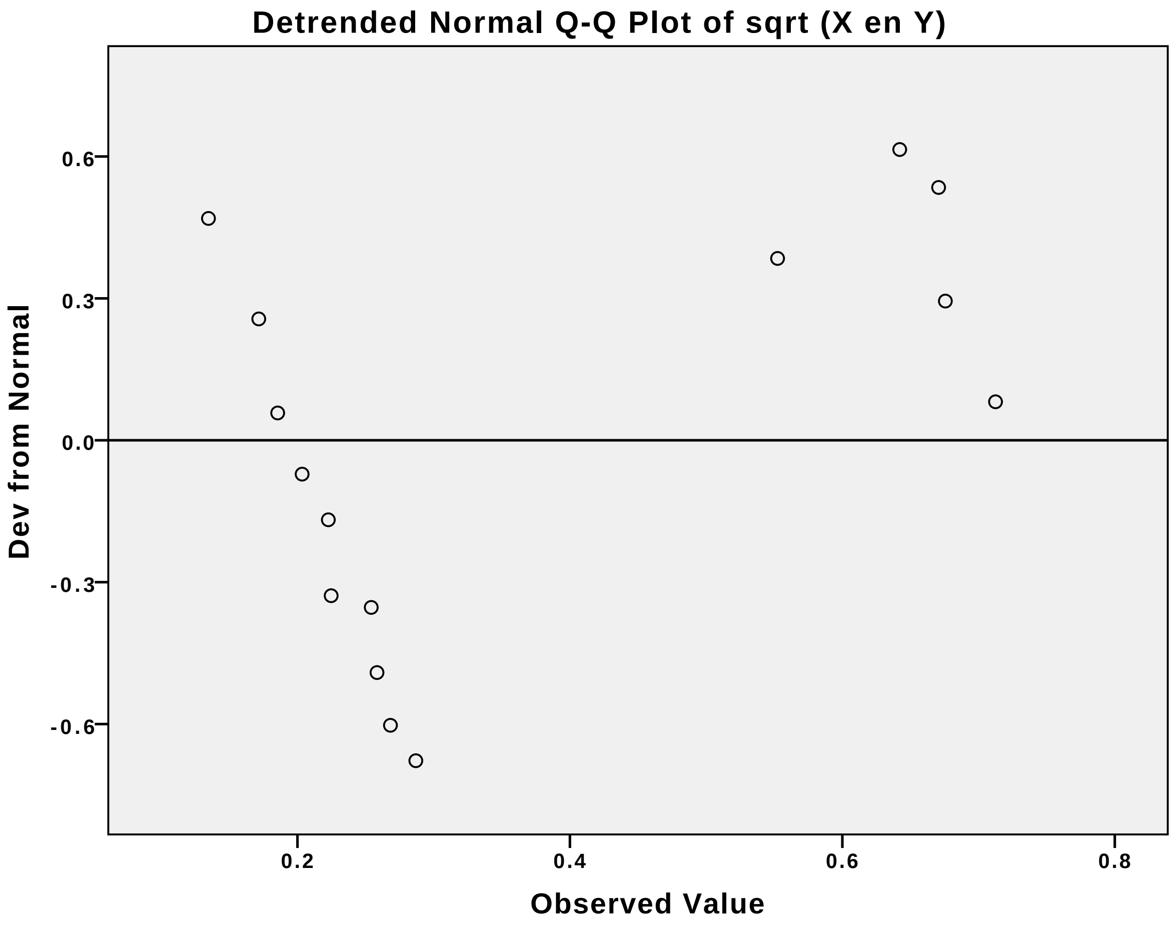

Detrended Normal Q-Q Plot of sqrt (X en Y)
Dev from Normal
0.6
0.3
0.0
-0.3
-0.6
0.2
0.4
0.6
0.8
Observed Value

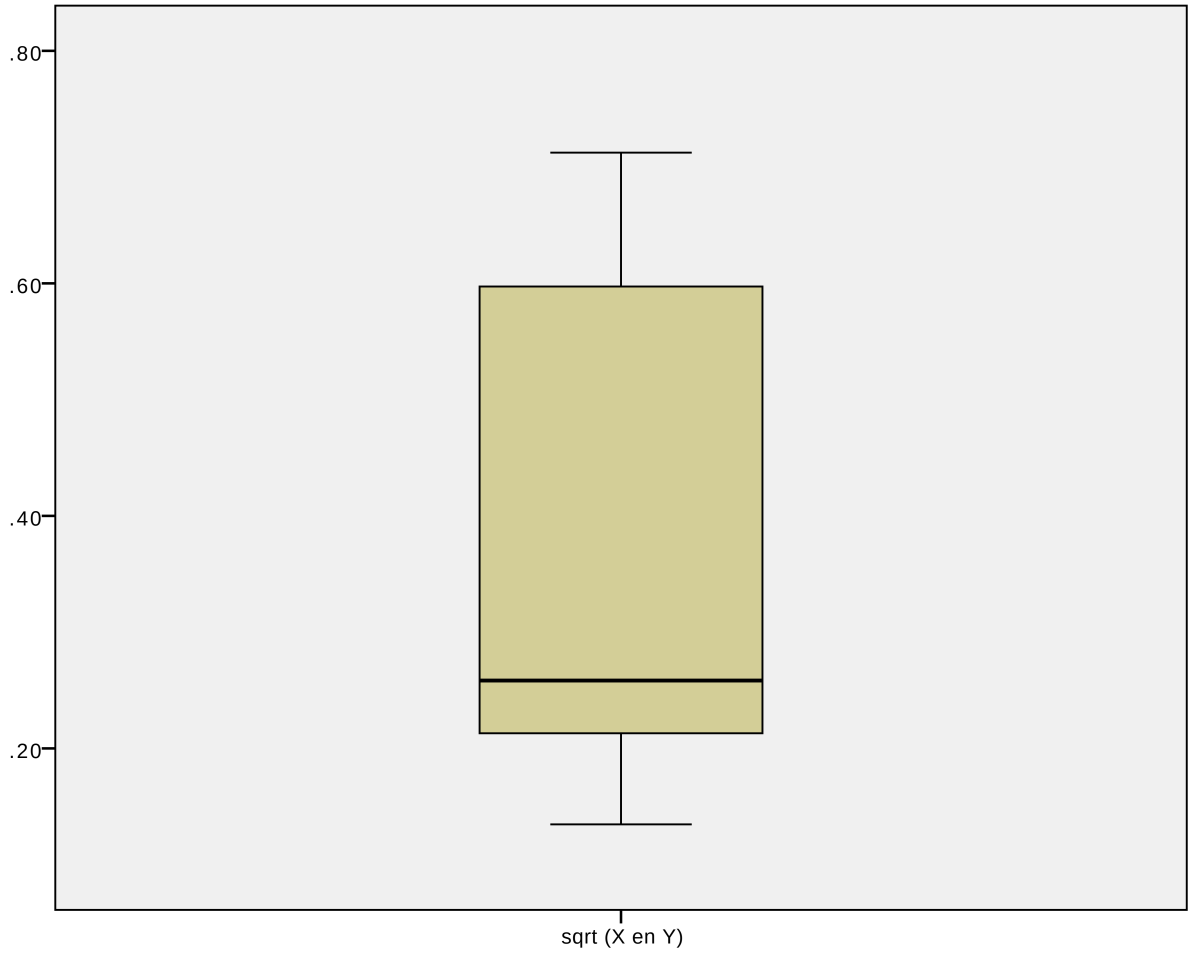


```
 *Nonparametric Tests: One Sample.
NPTESTS
  /ONESAMPLE TEST (sqrtXenY) WILCOXON(TESTVALUE=0)
  /MISSING SCOPE=ANALYSIS USERMISSING=EXCLUDE
  /CRITERIA ALPHA=0.05 CILEVEL=95.
```

Nonparametric Tests

Notes

Output Created		10-APR-2017 13:55:...
Comments		
Input	Active Dataset	DataSet3
	Filter	sqrtXenY < 0.94 (FILTER)
	Weight	<none>
	Split File	<none>
	N of Rows in Working Data File	15
Syntax		NPTESTS /ONESAMPLE TEST (sqrtXenY) WILCOXON (TESTVALUE=0) /MISSING SCOPE=ANALYSIS USERMISSING=EXCLUDE /CRITERIA ALPHA=0.05 CILEVEL=95.
Resources	Processor Time	00:00:00.08
	Elapsed Time	00:00:00.00

Hypothesis Test Summary

	Null Hypothesis	Test	Sig.	Decision
1	The median of sqrt (X en Y) equals 0.00.	One-Sample Wilcoxon Signed Rank Test	.001	Reject the null hypothesis.

Asymptotic significances are displayed. The significance level is .05.

```
DATASET ACTIVATE DataSet7.
EXAMINE VARIABLES=LATERAALTRANSVERSAALLENGTETRANSVERSAALDIEPTESAGGITAALL
ENGTESAGGITAAL
    DIEPTECORONAAL LATERAALCORRONAAL
  /PLOT BOXPLOT HISTOGRAM NPPLOT
  /COMPARE GROUPS
  /STATISTICS DESCRIPTIVES
  /CINTERVAL 95
  /MISSING LISTWISE
  /NOTOTAL.
```

Explore

Notes

Output Created		10-APR-2017 13:55:...
Comments		
Input	Active Dataset	DataSet7
	Filter	<none>
	Weight	<none>
	Split File	<none>
	N of Rows in Working Data File	18
Missing Value Handling	Definition of Missing	User-defined missing values for dependent variables are treated as missing.
	Cases Used	Statistics are based on cases with no missing values for any dependent variable or factor used.
Syntax		EXAMINE VARIABLES=LATERAALTRANSVERSAAL LENGTETRANSVERSAAL DIEPTESAGGITAAL LENGTESAGGITAAL DIEPTECORONAAL LATERAALCORRONAAL /PLOT BOXPLOT HISTOGRAM NPPLOT /COMPARE GROUPS /STATISTICS DESCRIPTIVES /CINTERVAL 95 /MISSING LISTWISE /NOTOTAL.
Resources	Processor Time	00:00:03.34
	Elapsed Time	00:00:04.00

[DataSet7]

Case Processing Summary

	Cases					
	Valid		Missing		Total	
	N	Percent	N	Percent	N	Percent
LATERAAL - TRANSVERSAAL	15	83.3%	3	16.7%	18	100.0%
LENGTE-TRANSVERSAAL	15	83.3%	3	16.7%	18	100.0%
DIEPTE - SAGGITAAL	15	83.3%	3	16.7%	18	100.0%
LENGTE - SAGGITAAL	15	83.3%	3	16.7%	18	100.0%
DIEPTE-CORONAAL	15	83.3%	3	16.7%	18	100.0%
LATERAAL-CORRONAAL	15	83.3%	3	16.7%	18	100.0%

Descriptives

			Statistic	Std. Error
LATERAAL - TRANSVERSAAL	Mean		.1497	.03124
	95% Confidence Interval for Mean	Lower Bound	.0827	
		Upper Bound	.2168	
	5% Trimmed Mean		.1341	
	Median		.1340	
	Variance		.015	
	Std. Deviation		.12100	
	Minimum		.05	
	Maximum		.53	
	Range		.48	
	Interquartile Range		.10	
	Skewness		2.426	.580
	Kurtosis		7.054	1.121
LENGTE-TRANSVERSAAL	Mean		.3147	.05466
	95% Confidence Interval for Mean	Lower Bound	.1975	
		Upper Bound	.4319	
	5% Trimmed Mean		.3076	
	Median		.2527	
	Variance		.045	
	Std. Deviation		.21168	
	Minimum		.08	
	Maximum		.68	
	Range		.60	
	Interquartile Range		.43	
	Skewness		.736	.580
	Kurtosis		-1.070	1.121
DIEPTE - SAGGITAAL	Mean		1.7032	.06134
	95% Confidence Interval for Mean	Lower Bound	1.5716	
		Upper Bound	1.8348	
	5% Trimmed Mean		1.7028	
	Median		1.6700	
	Variance		.056	
	Std. Deviation		.23755	
	Minimum		1.30	
	Maximum		2.12	
	Range		.82	
	Interquartile Range		.33	
	Skewness		.187	.580
	Kurtosis		-.666	1.121
LENGTE - SAGGITAAL	Mean		.3524	.04547
	95% Confidence Interval for Mean	Lower Bound	.2549	
		Upper Bound	.4500	
	5% Trimmed Mean		.3490	
	Median		.2563	
	Variance		.031	
	Std. Deviation		.17612	
	Minimum		.11	
	Maximum		.66	

Descriptives

			Statistic	Std. Error
	Range		.55	
	Interquartile Range		.30	
	Skewness		.543	.580
	Kurtosis		-1.206	1.121
DIEPTE-CORONAAL	Mean		1.7362	.05859
	95% Confidence Interval for Mean	Lower Bound	1.6105	
		Upper Bound	1.8619	
	5% Trimmed Mean		1.7395	
	Median		1.6567	
	Variance		.051	
	Std. Deviation		.22694	
	Minimum		1.33	
	Maximum		2.08	
	Range		.75	
	Interquartile Range		.37	
	Skewness		.083	.580
	Kurtosis		-.932	1.121
LATERAAL-CORRONAAL	Mean		.1387	.03396
	95% Confidence Interval for Mean	Lower Bound	.0659	
		Upper Bound	.2116	
	5% Trimmed Mean		.1219	
	Median		.0943	
	Variance		.017	
	Std. Deviation		.13151	
	Minimum		.03	
	Maximum		.55	
	Range		.52	
	Interquartile Range		.10	
	Skewness		2.497	.580
	Kurtosis		7.064	1.121

Tests of Normality

	Kolmogorov-Smirnov[a]			Shapiro-Wilk		
	Statistic	df	Sig.	Statistic	df	Sig.
LATERAAL - TRANSVERSAAL	.256	15	.009	.731	15	.001
LENGTE-TRANSVERSAAL	.267	15	.005	.850	15	.018
DIEPTE - SAGGITAAL	.130	15	.200*	.977	15	.946
LENGTE - SAGGITAAL	.241	15	.019	.890	15	.067
DIEPTE-CORONAAL	.170	15	.200*	.944	15	.441
LATERAAL-CORRONAAL	.221	15	.048	.709	15	.000

*. This is a lower bound of the true significance.

a. Lilliefors Significance Correction

LATERAAL - TRANSVERSAAL

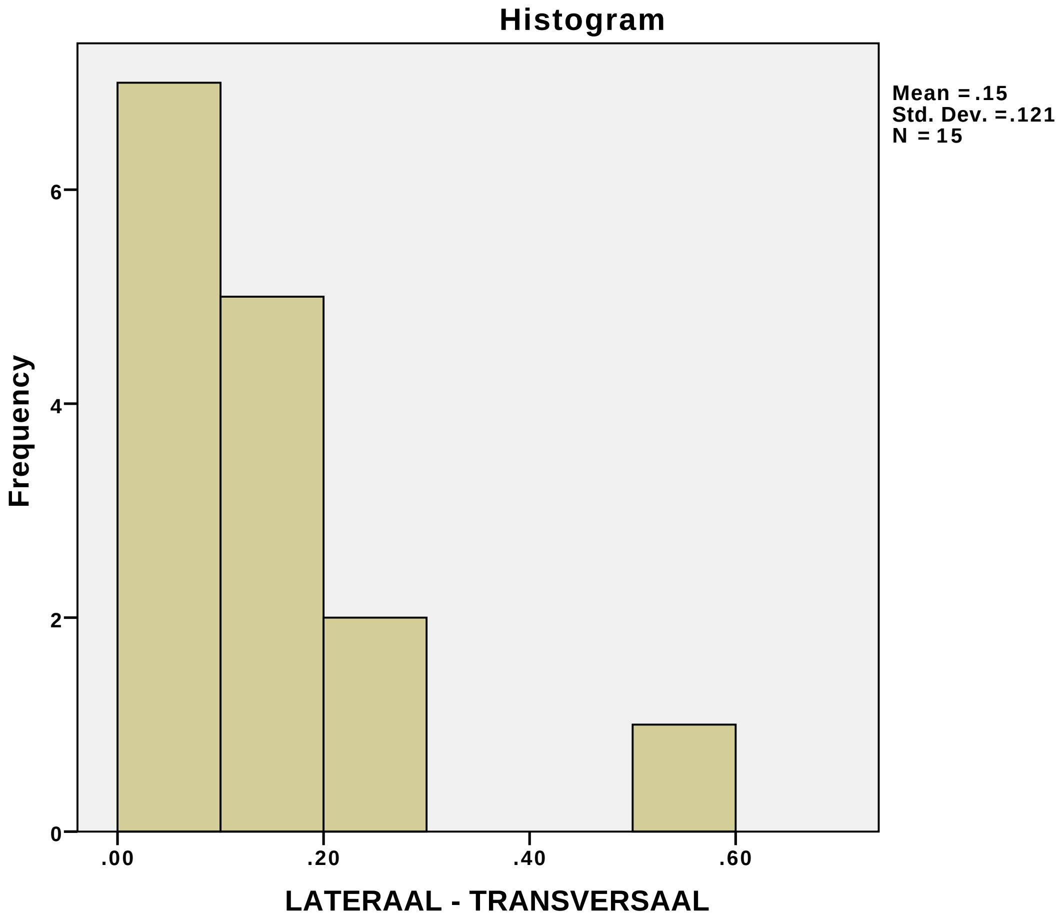

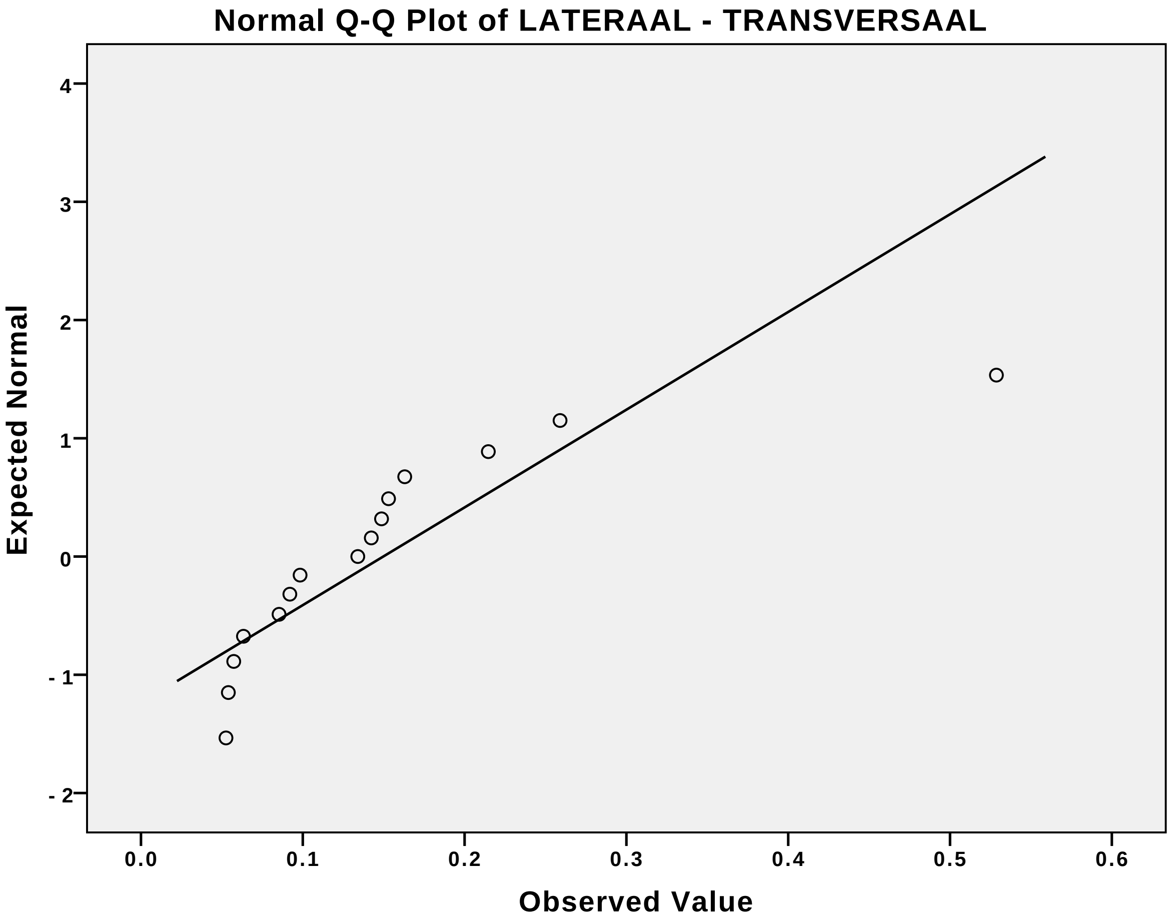
Normal Q-Q Plot of LATERAAL - TRANSVERSAAL
Expected Normal
4
3
2
1
0
- 1
- 2
0.0
0.1
0.2
0.3
0.4
0.5
0.6
Observed Value

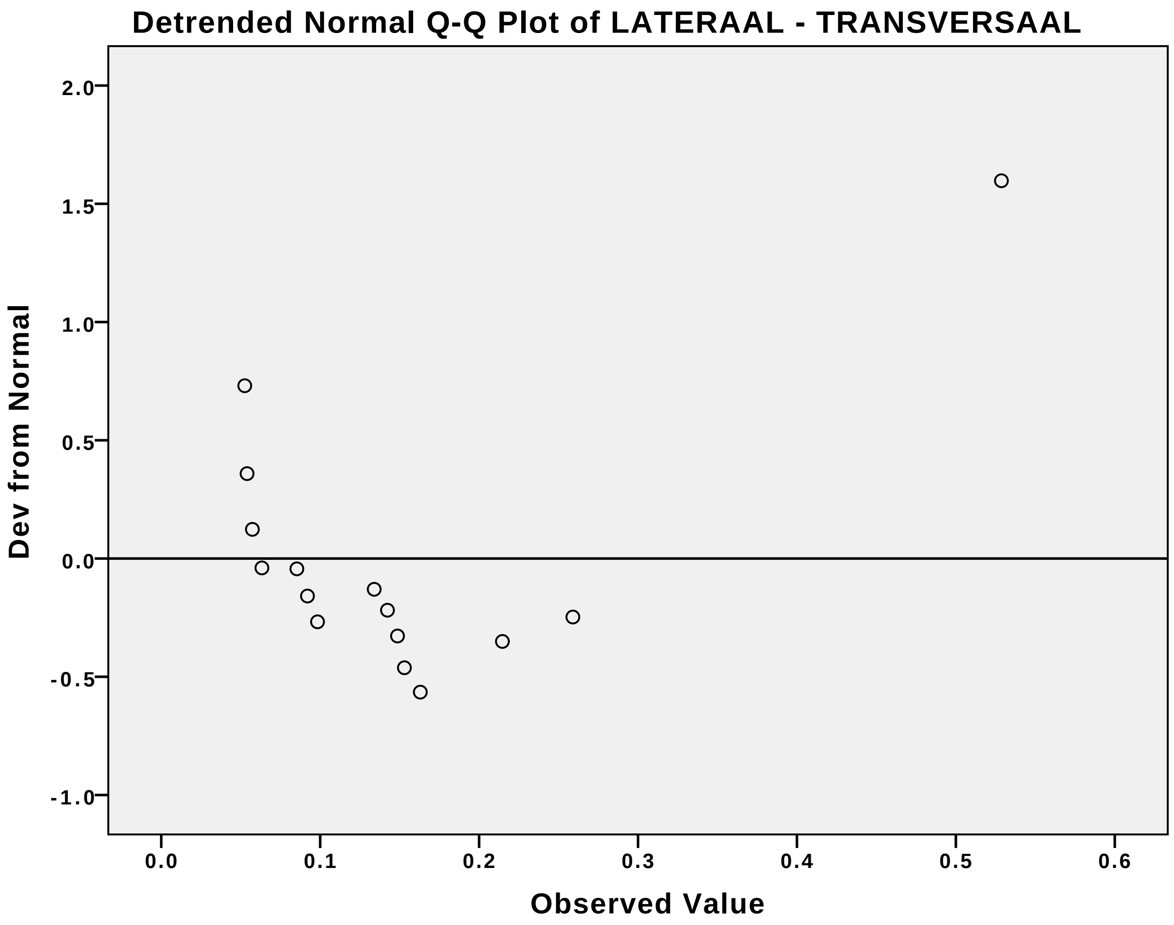
Detrended Normal Q-Q Plot of LATERAAL - TRANSVERSAAL
Dev from Normal
2.0
1.5
1.0
0.5
0.0
-0.5
-1.0
0.0
0.1
0.2
0.3
0.4
0.5
0.6
Observed Value

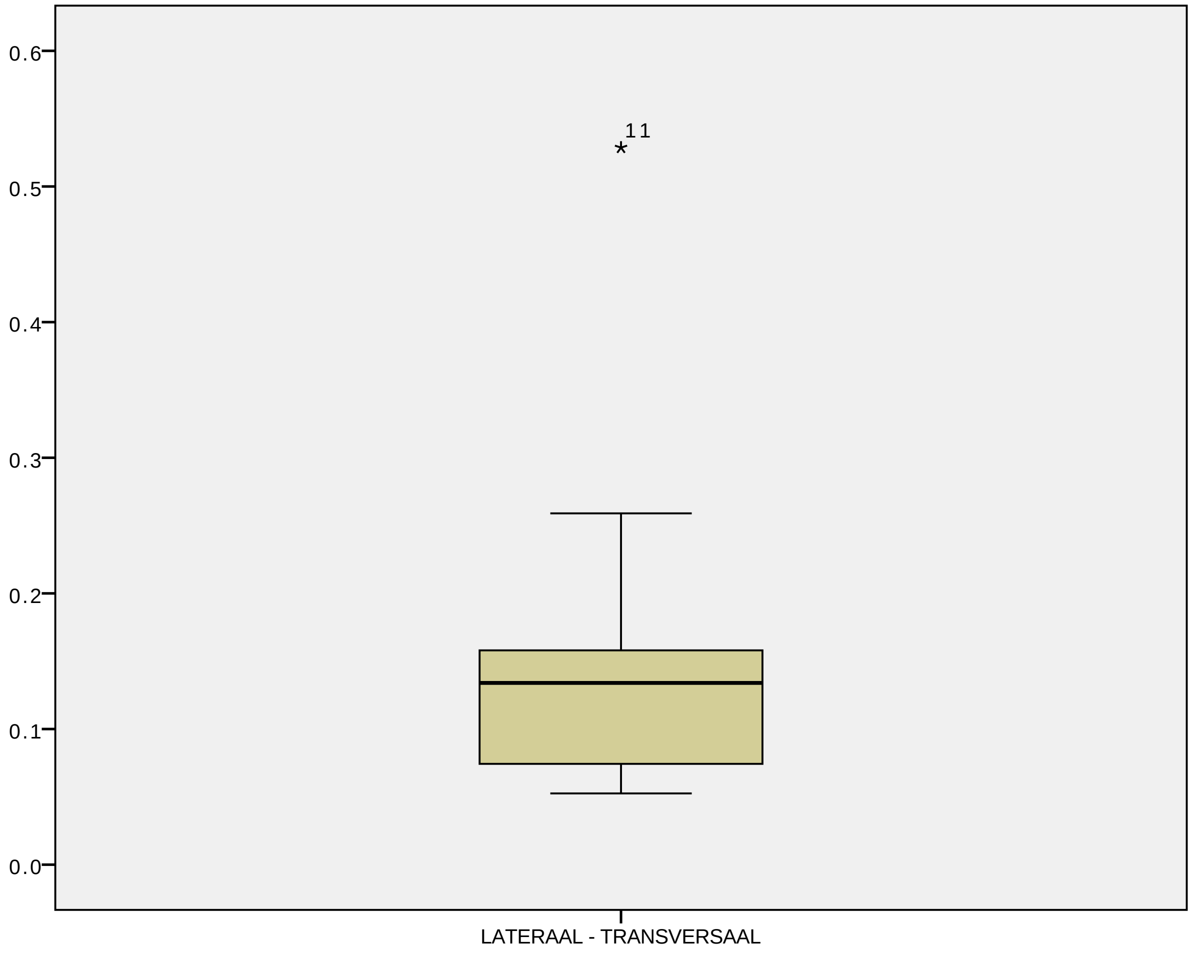

LENGTE-TRANSVERSAAL

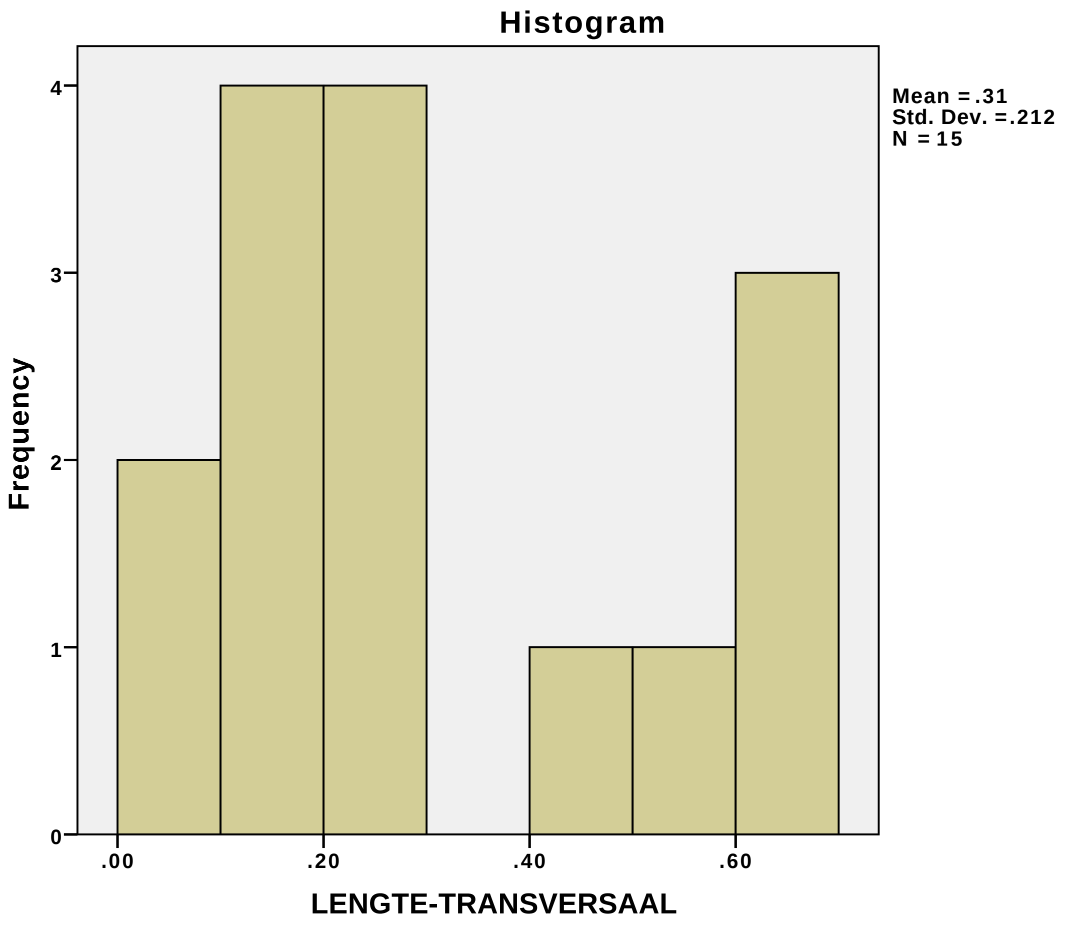
Histogram
Mean = .31
Std. Dev. =.212
N = 15
Frequency
0
1
2
3
4
.00
.20
.40
.60
LENGTE-TRANSVERSAAL

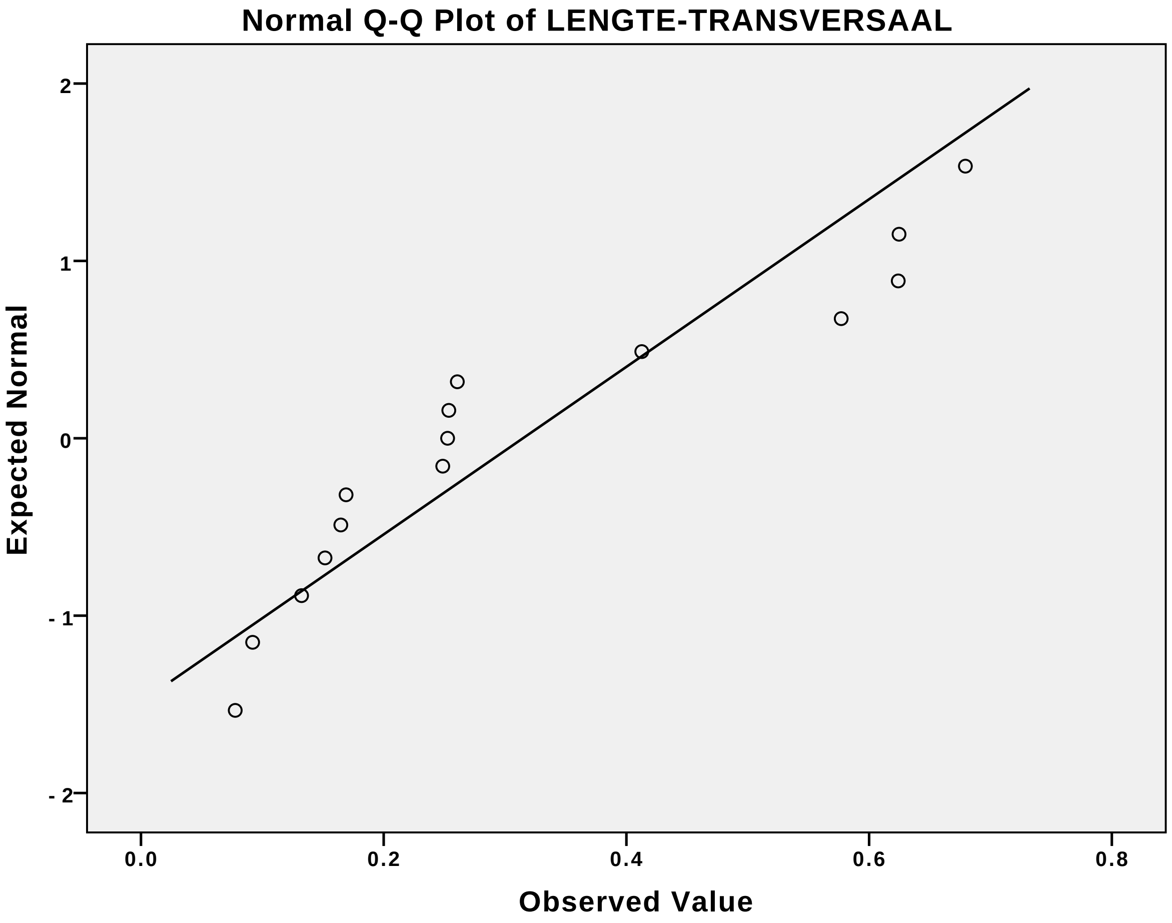
Normal Q-Q Plot of LENGTE-TRANSVERSAAL
Expected Normal
2
1
0
- 1
- 2
0.0
0.2
0.4
0.6
0.8
Observed Value

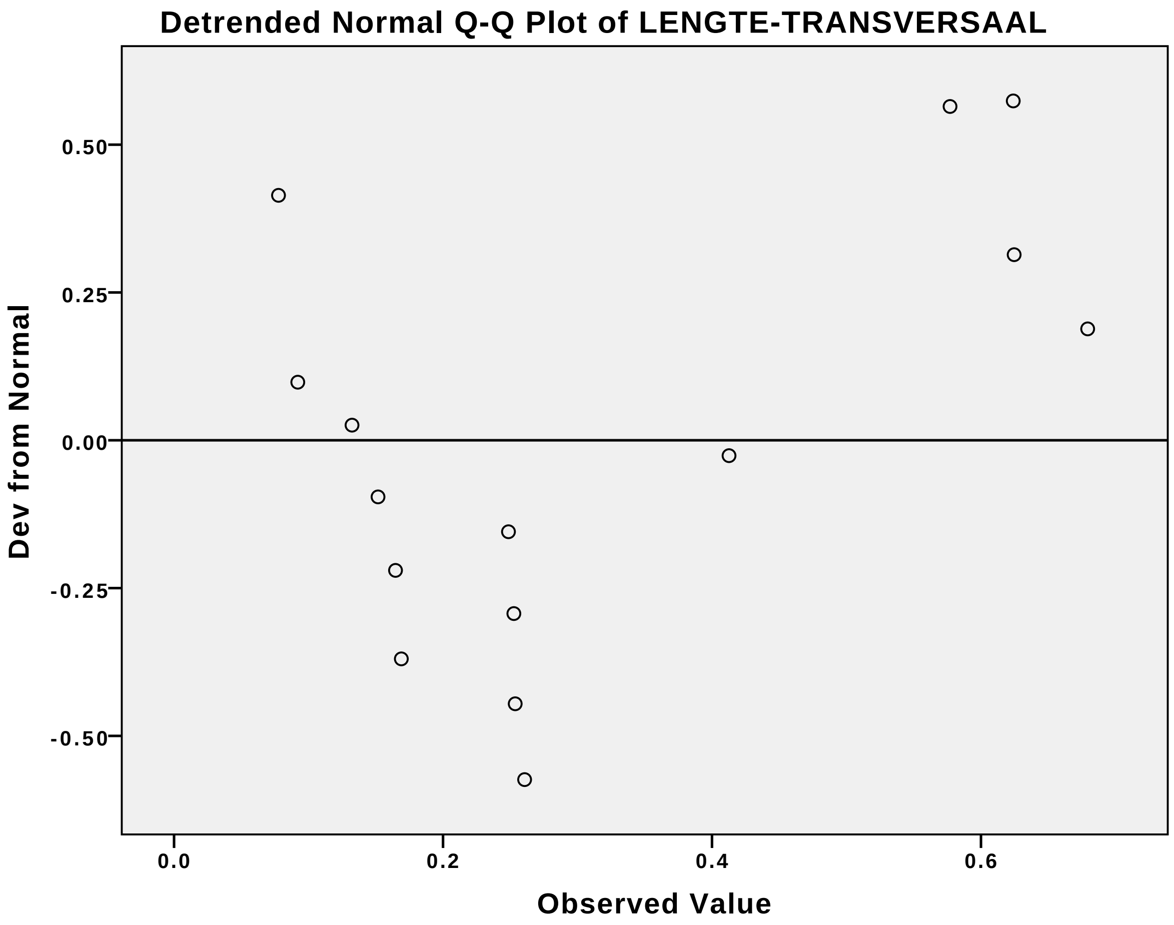
Detrended Normal Q-Q Plot of LENGTE-TRANSVERSAAL
Dev from Normal
0.50
0.25
0.00
-0.25
-0.50
0.0
0.2
0.4
0.6
Observed Value

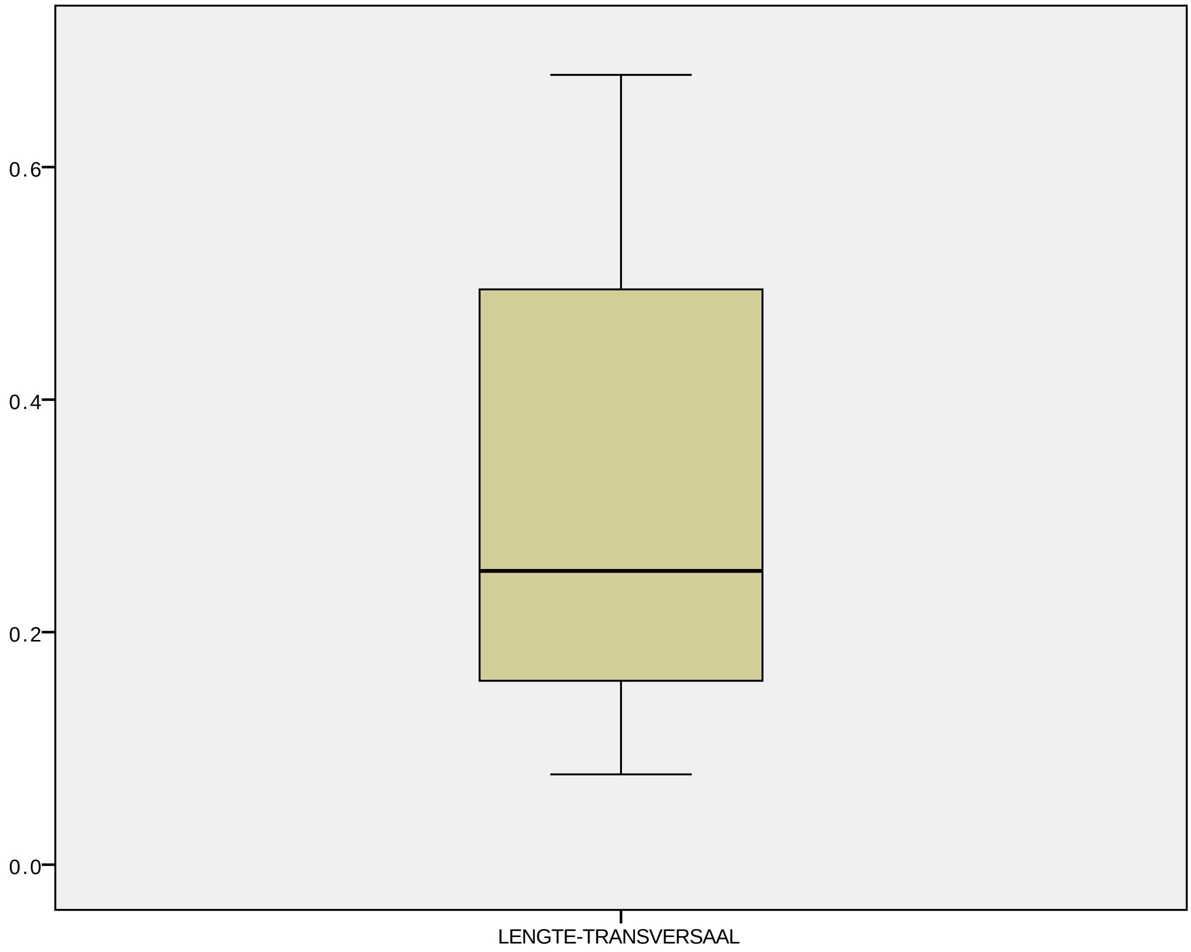

DIEPTE - SAGGITAAL

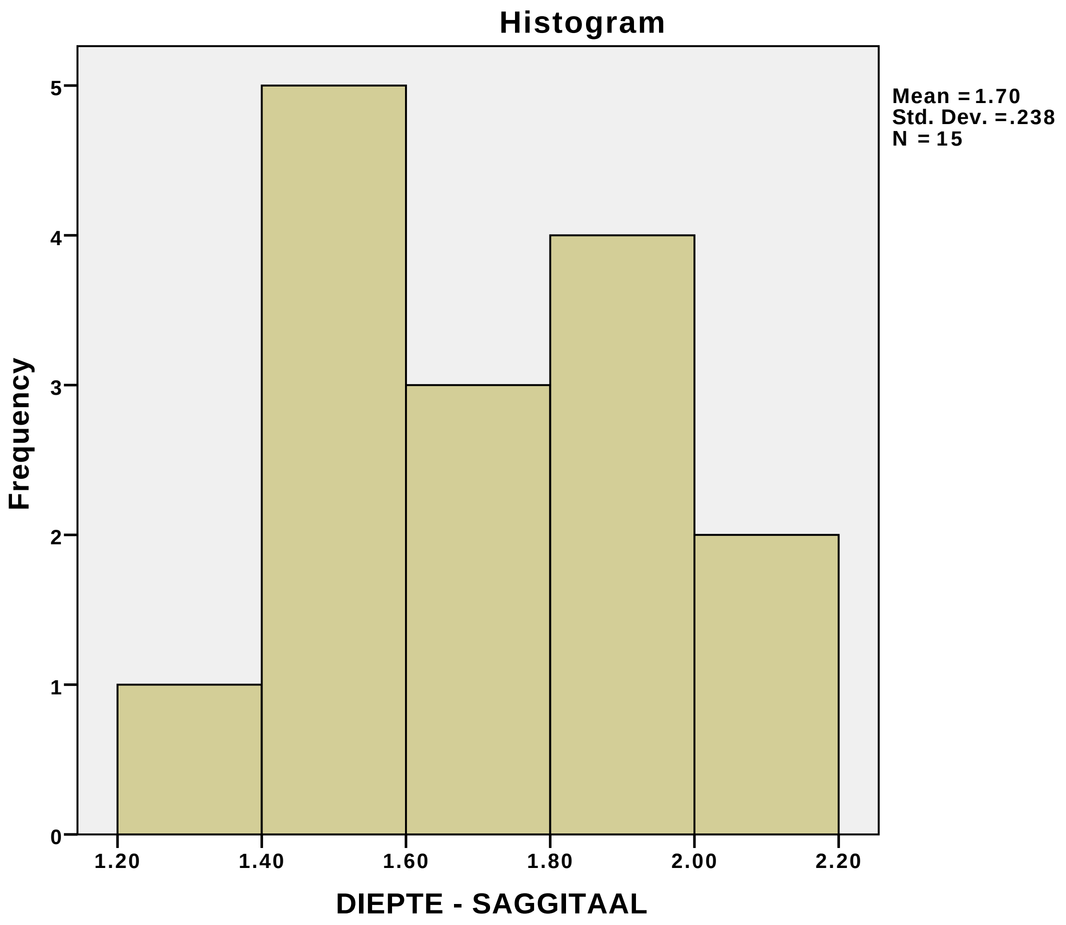
Histogram
Mean =1.70
Std. Dev. =.238
N =15
Frequency
0
1
2
3
4
5
1.20
1.40
1.60
1.80
2.00
2.20
DIEPTE - SAGGITAAL

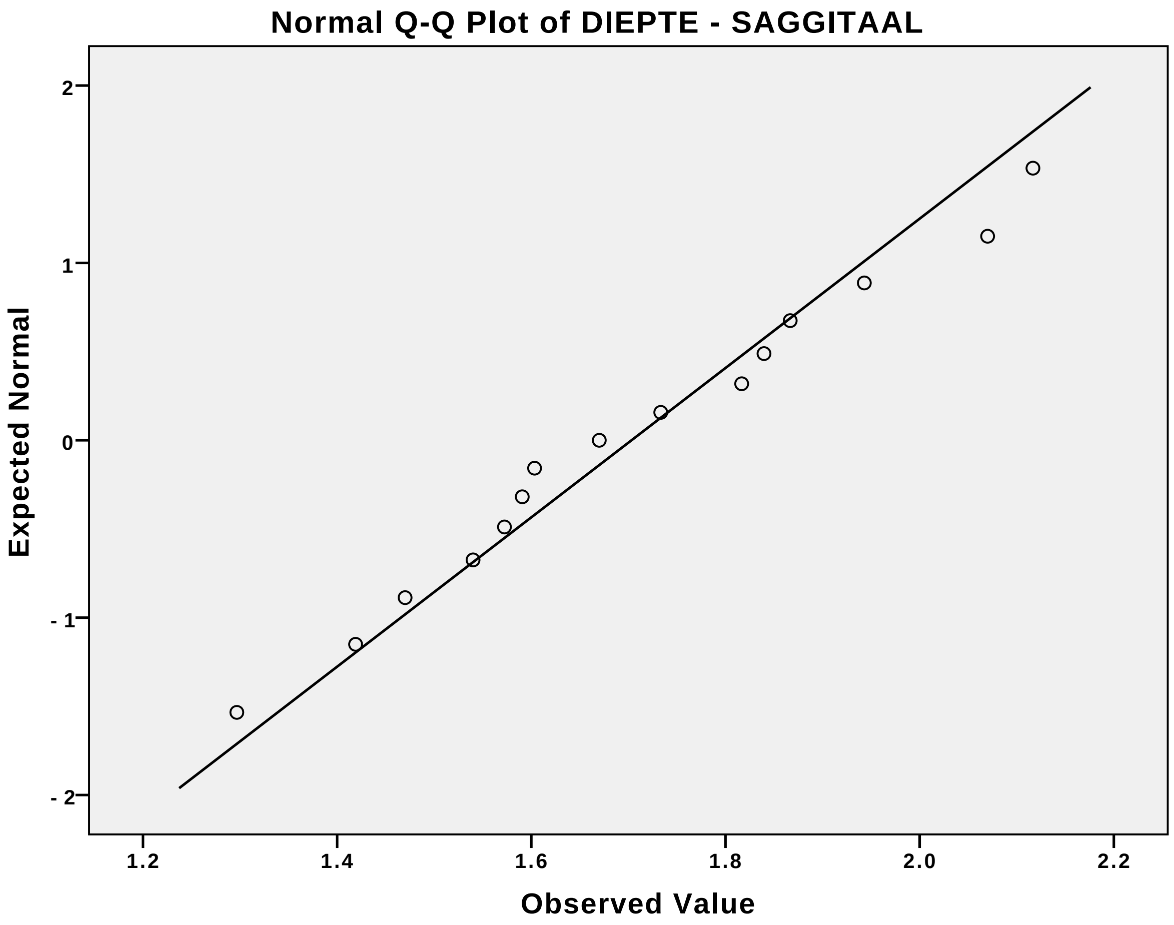
Normal Q-Q Plot of DIEPTE - SAGGITAAL
Expected Normal
2
1
0
- 1
- 2
1.2
1.4
1.6
1.8
2.0
2.2
Observed Value

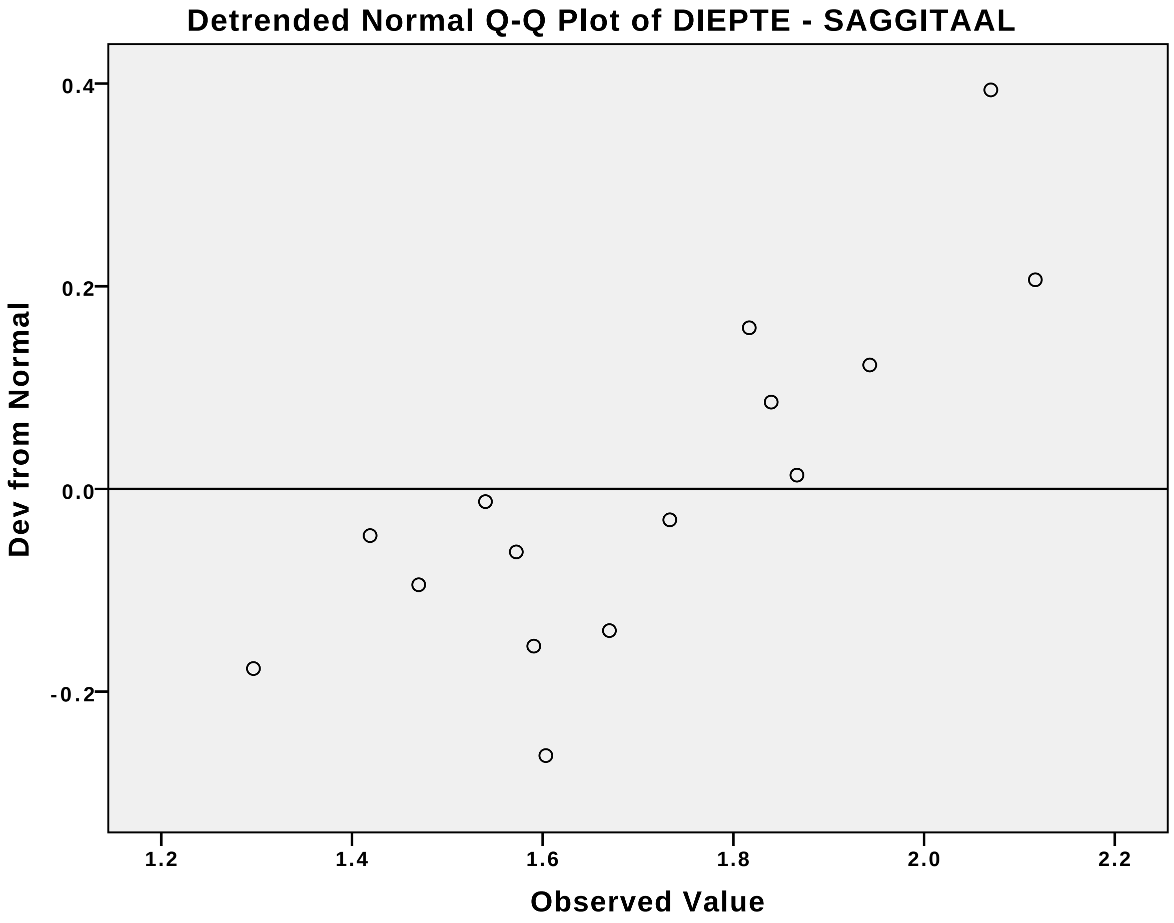

Detrended Normal Q-Q Plot of DIEPTE - SAGGITAAL
0.4
0.2
0.0
-0.2
Dev from Normal
1.2
1.4
1.6
1.8
2.0
2.2
Observed Value

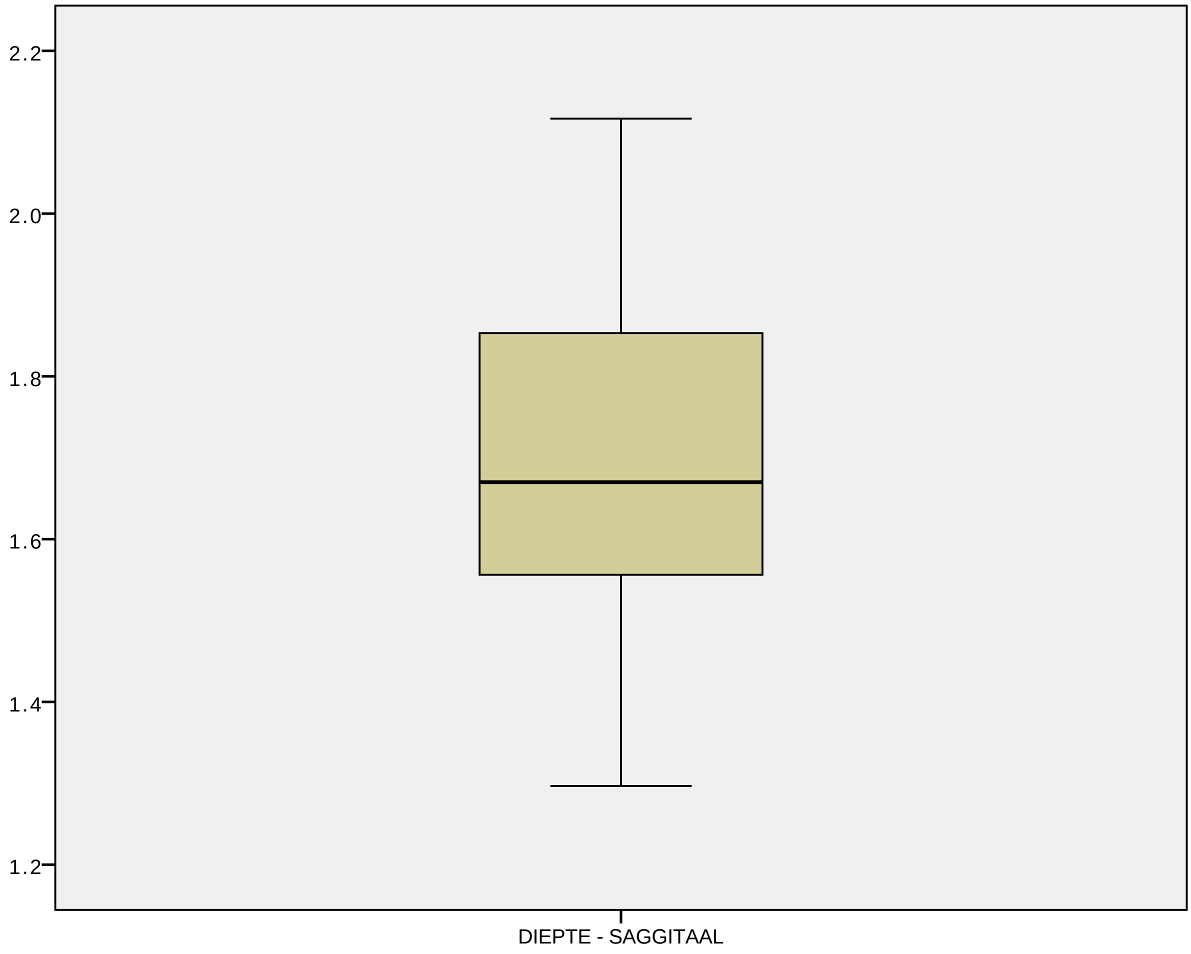

LENGTE - SAGGITAAL

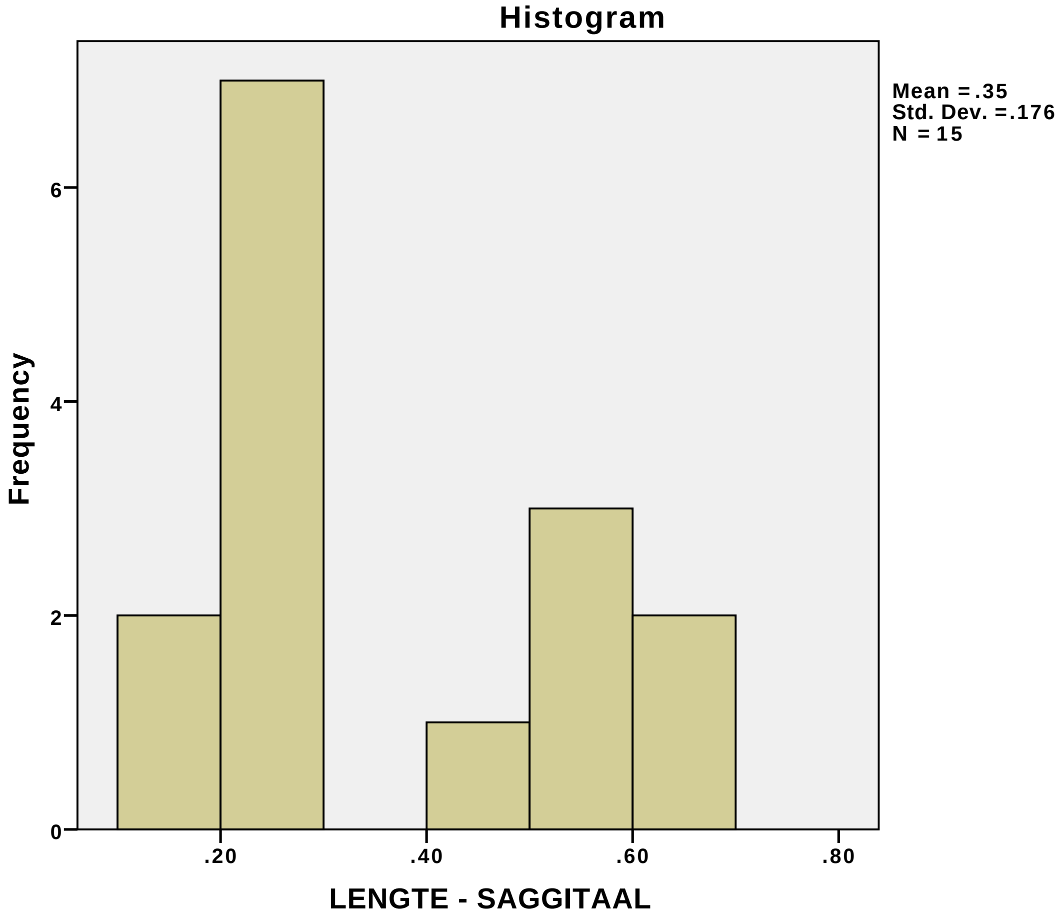

Histogram
Mean = .35
Std. Dev. = .176
N = 15
Frequency
0
2
4
6
.20
.40
.60
.80
LENGTE - SAGGITAAL

Normal Q-Q Plot of LENGTE - SAGGITAAL

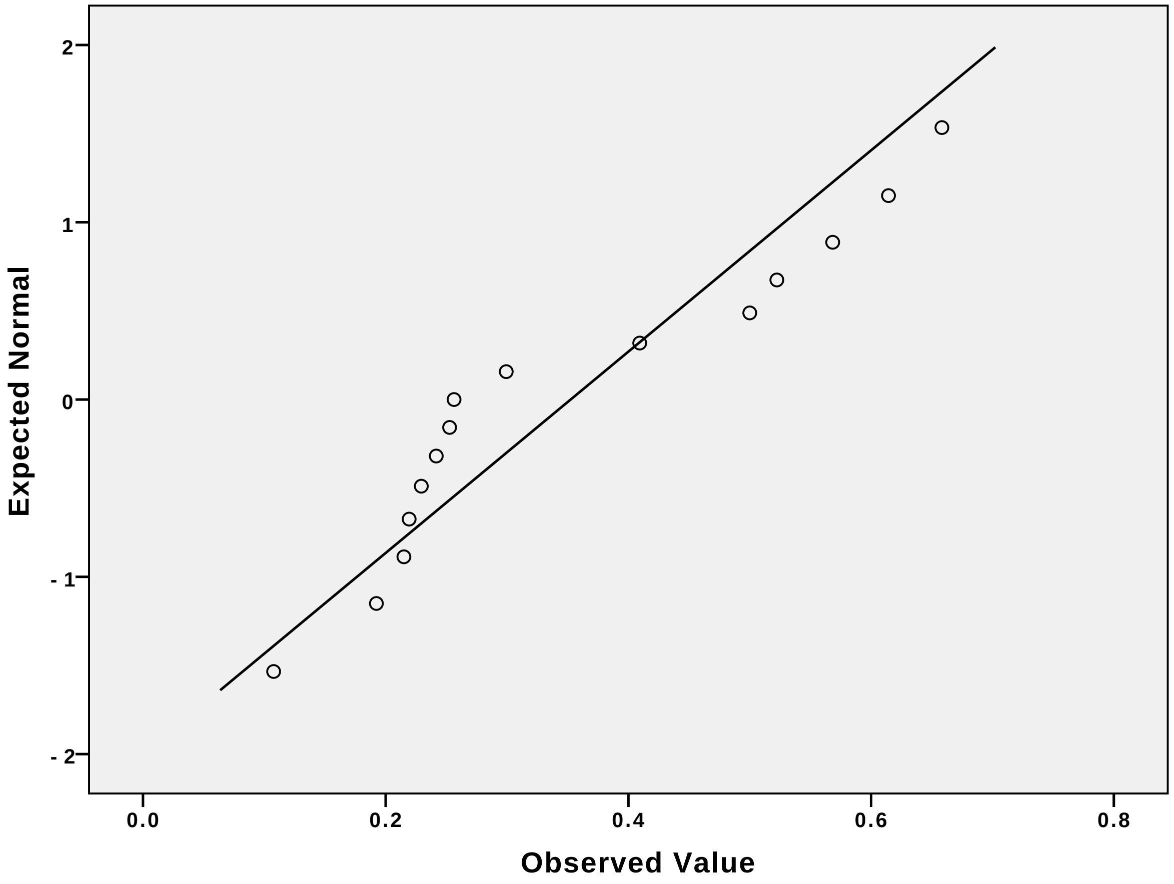

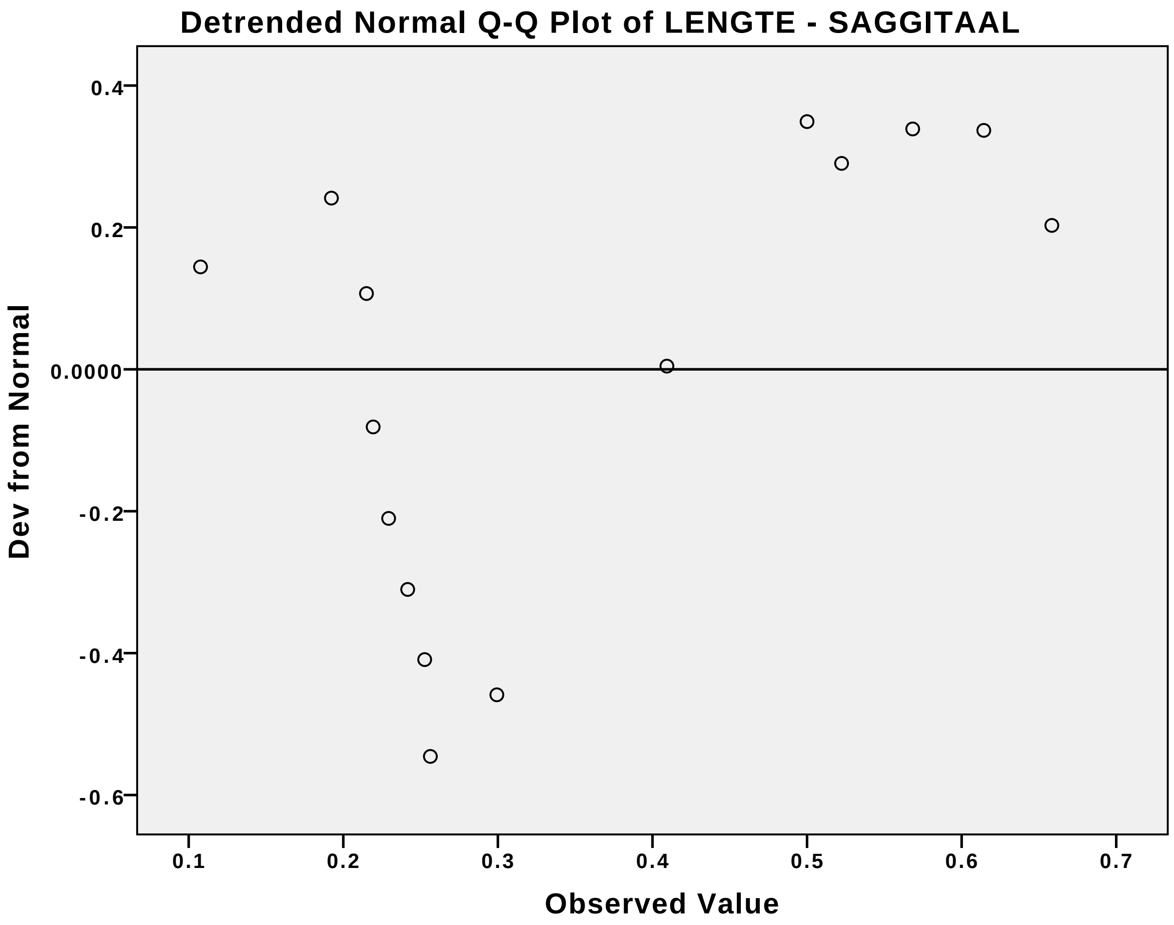
Detrended Normal Q-Q Plot of LENGTE - SAGGITAAL
Dev from Normal
0.4
0.2
0.0000
-0.2
-0.4
-0.6
0.1
0.2
0.3
0.4
0.5
0.6
0.7
Observed Value

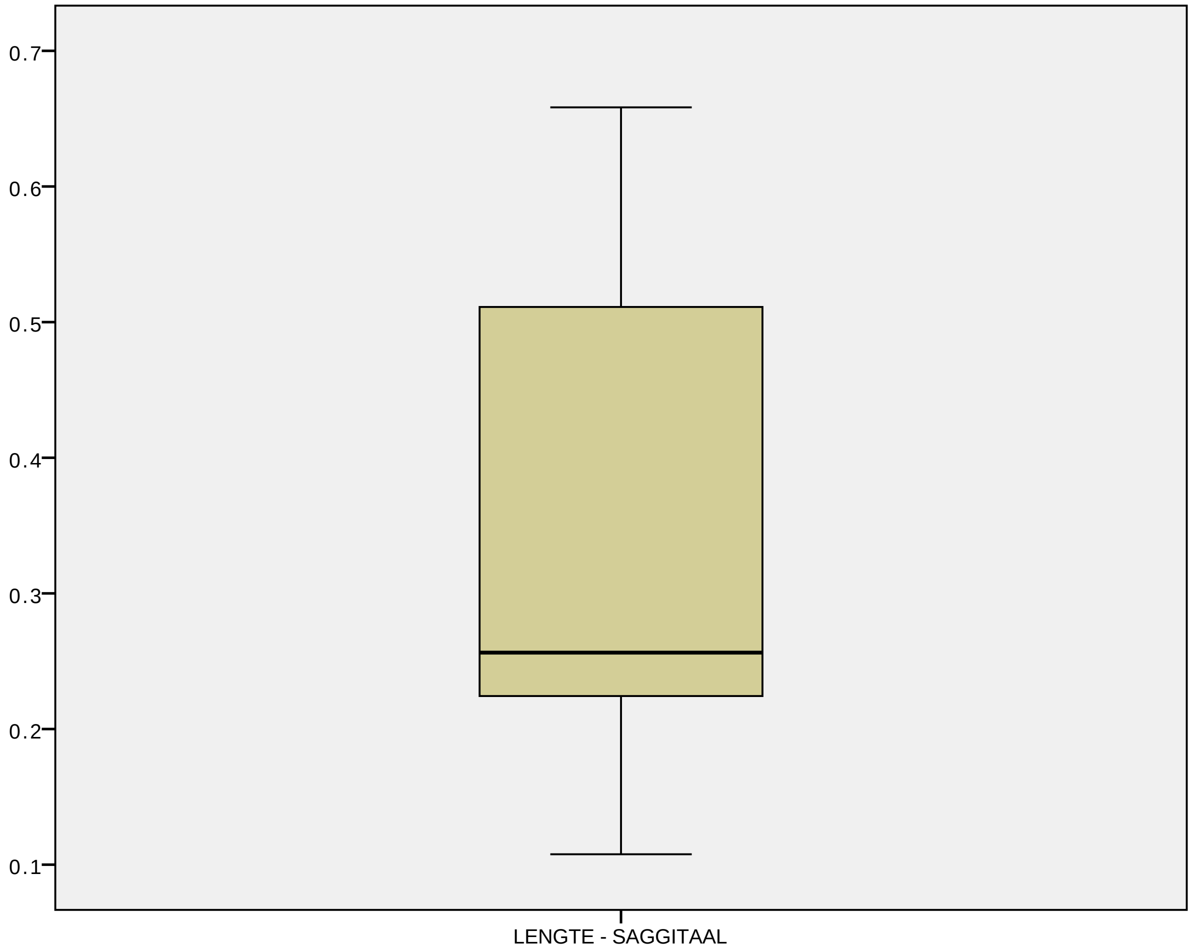

DIEPTE-CORONAAL

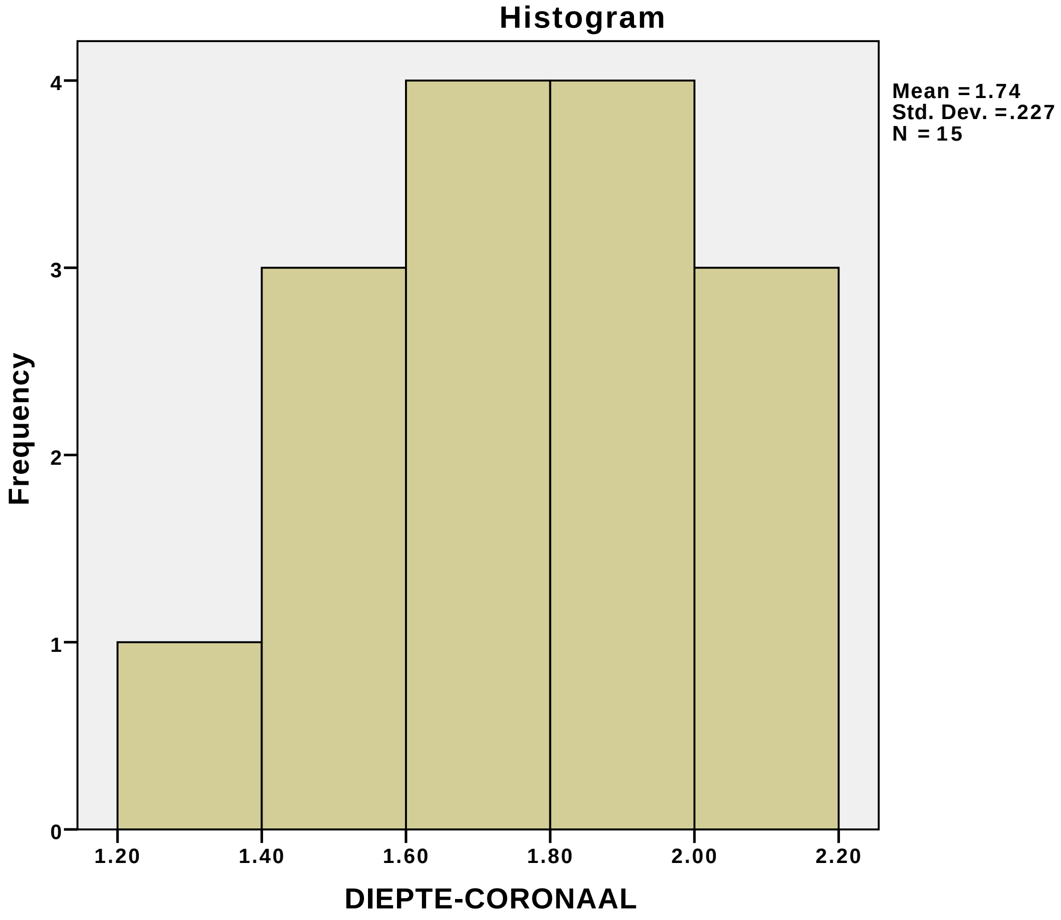
Histogram
Mean =1.74
Std. Dev. =.227
N =15
Frequency
0
1
2
3
4
1.20
1.40
1.60
1.80
2.00
2.20
DIEPTE-CORONAAL

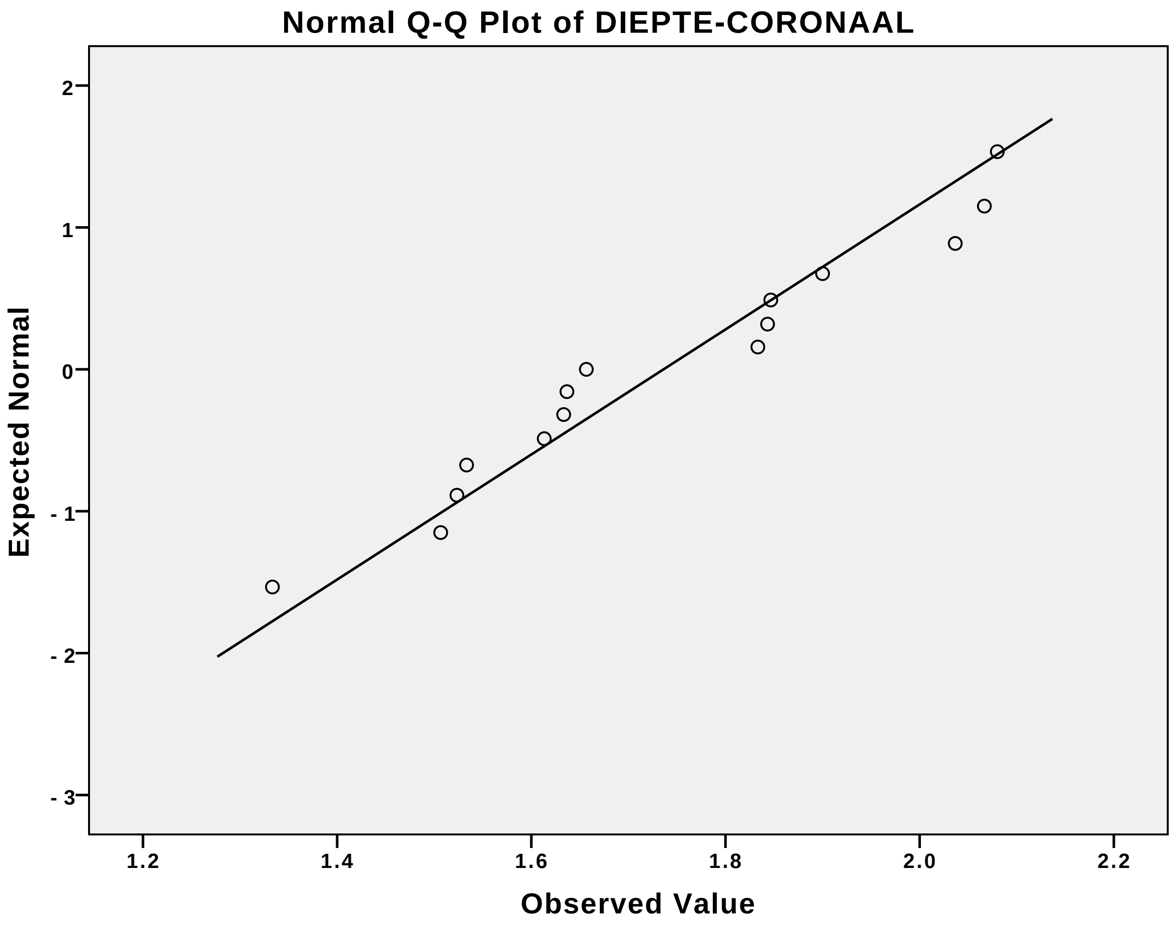
Normal Q-Q Plot of DIEPTE-CORONAAL
Expected Normal
Observed Value
2
1
0
- 1
- 2
- 3
1.2
1.4
1.6
1.8
2.0
2.2

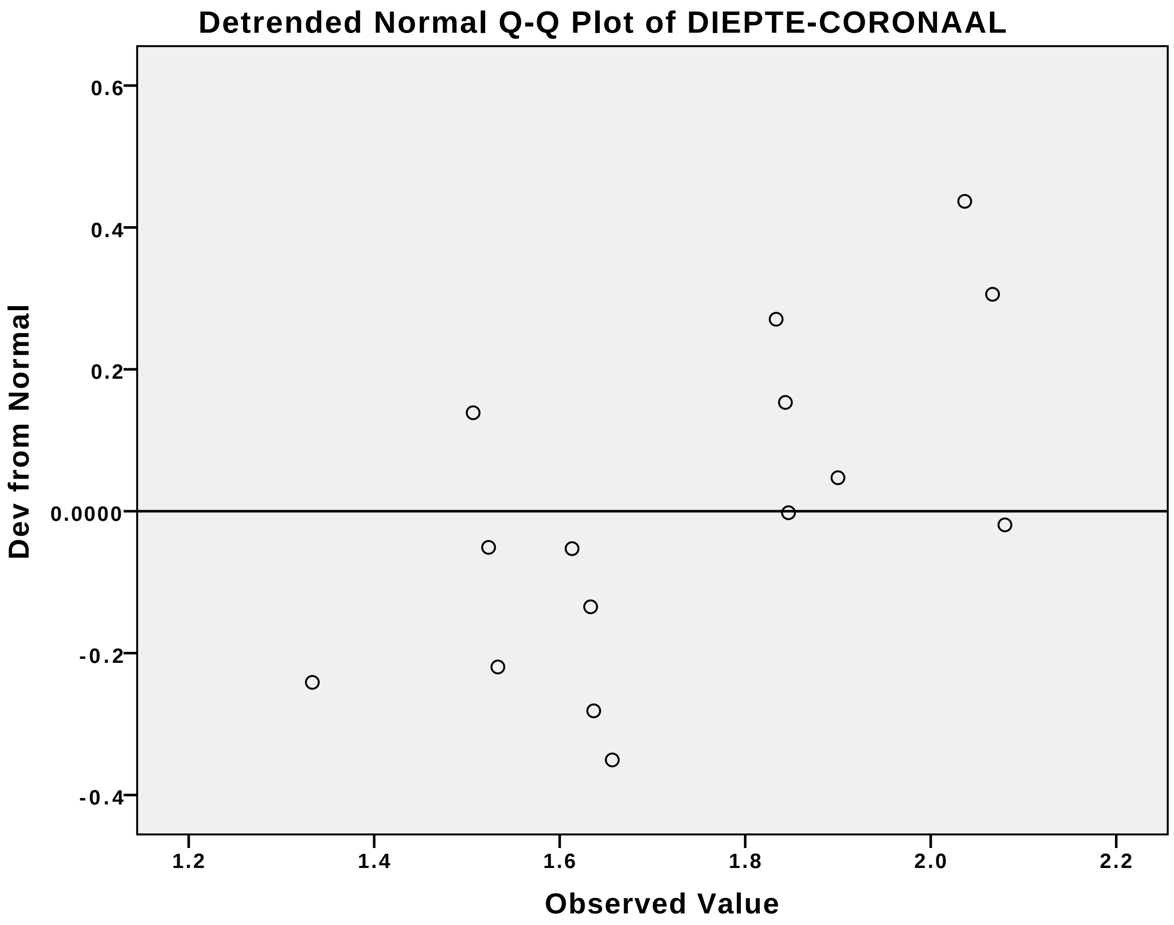
Detrended Normal Q-Q Plot of DIEPTE-CORONAAL
Dev from Normal
0.6
0.4
0.2
0.0000
-0.2
-0.4
1.2
1.4
1.6
1.8
2.0
2.2
Observed Value

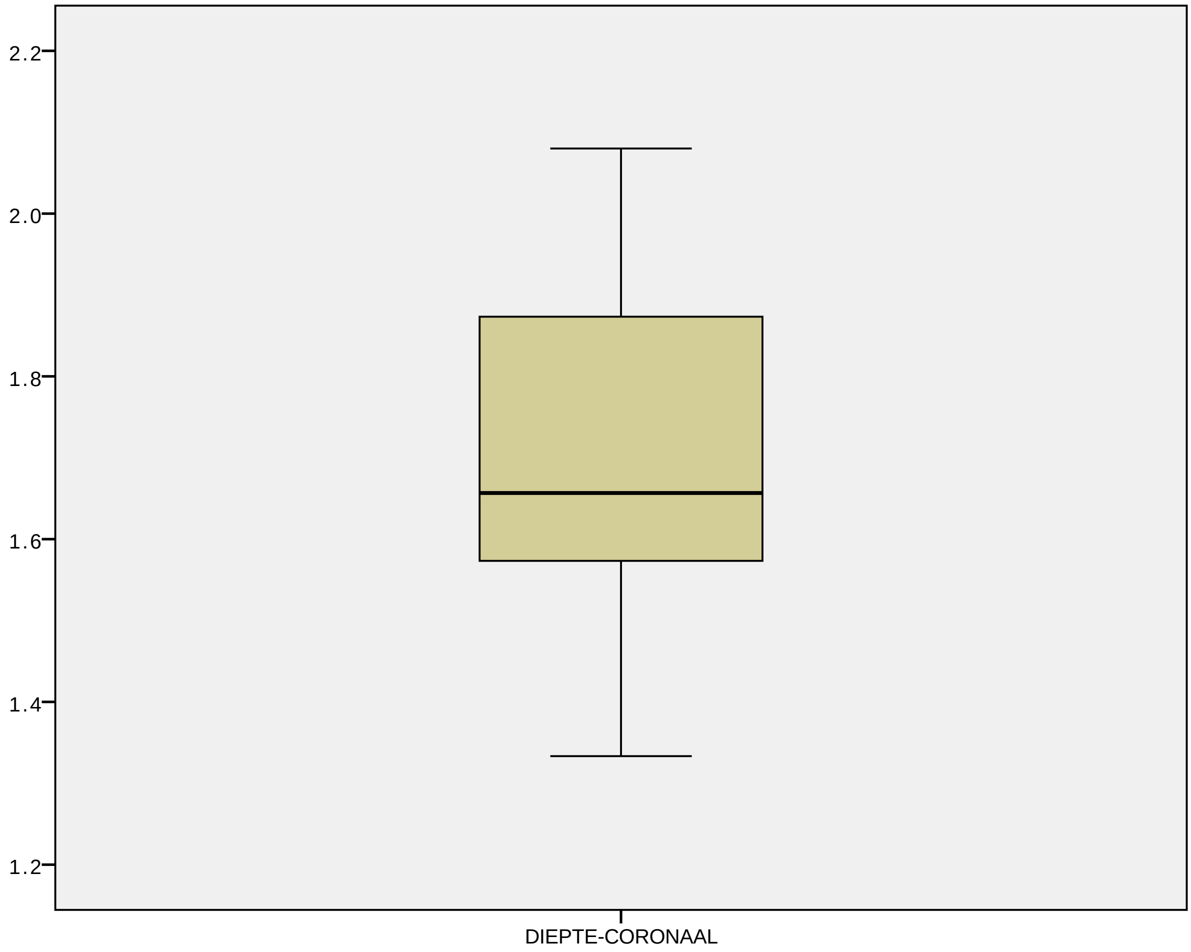

LATERAAL-CORRONAAL

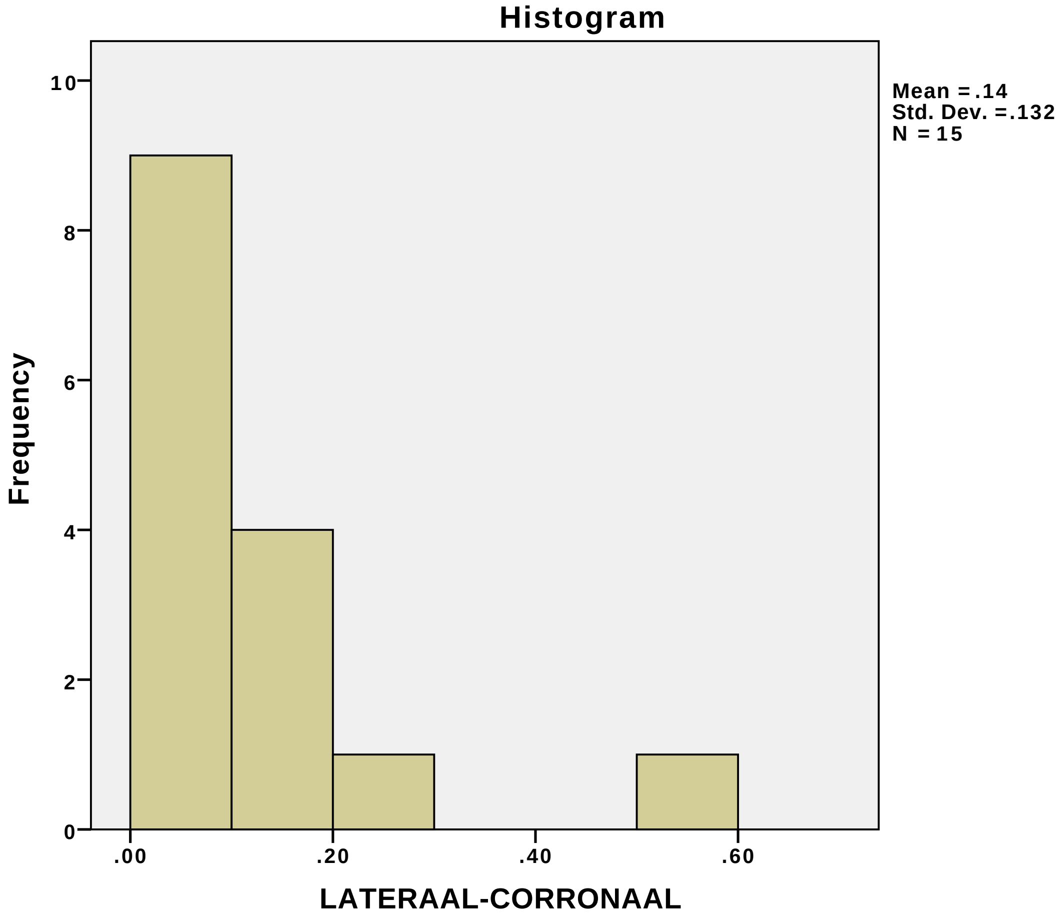
Histogram
Mean =.14
Std. Dev. =.132
N =15
Frequency
10
8
6
4
2
0
.00
.20
.40
.60
LATERAAL-CORRONAAL

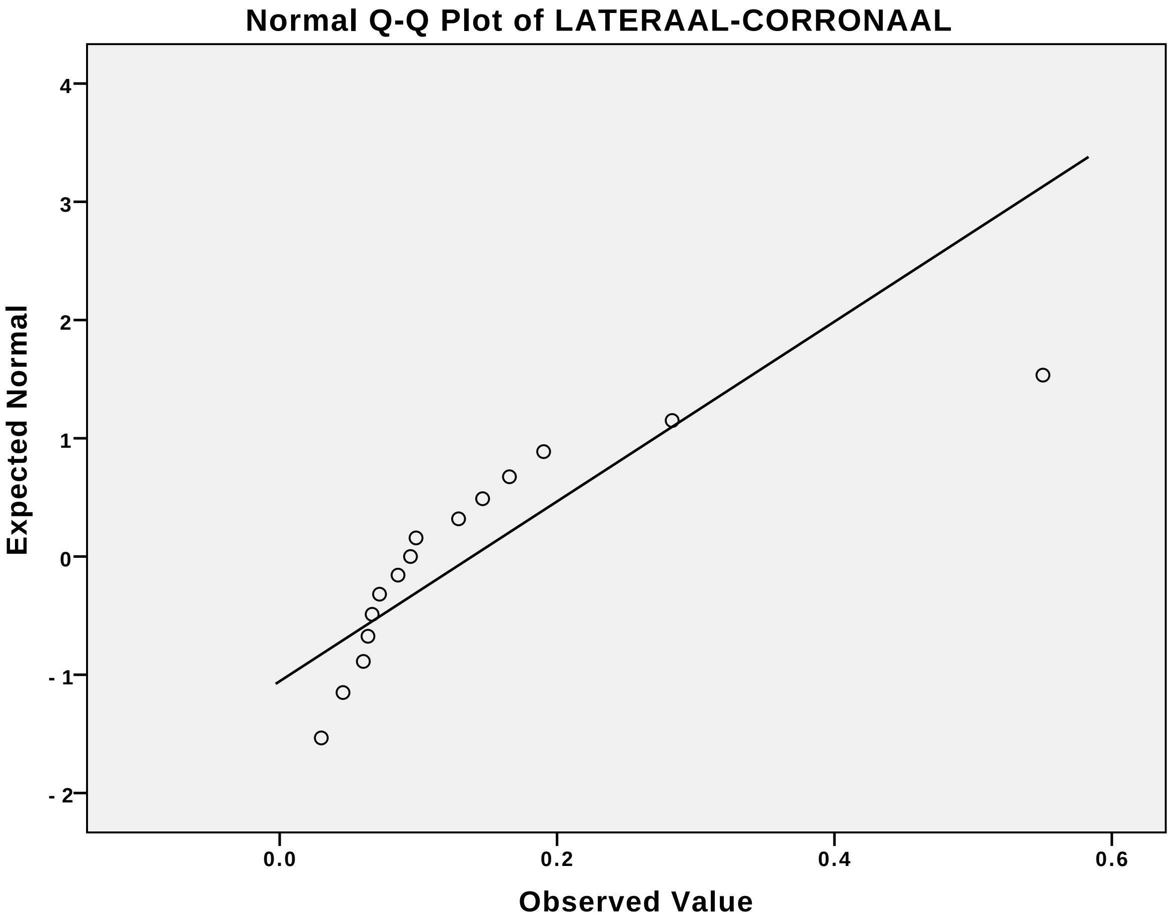
Normal Q-Q Plot of LATERAAL-CORRONAAL
Expected Normal
4
3
2
1
0
- 1
- 2
0.0
0.2
0.4
0.6
Observed Value

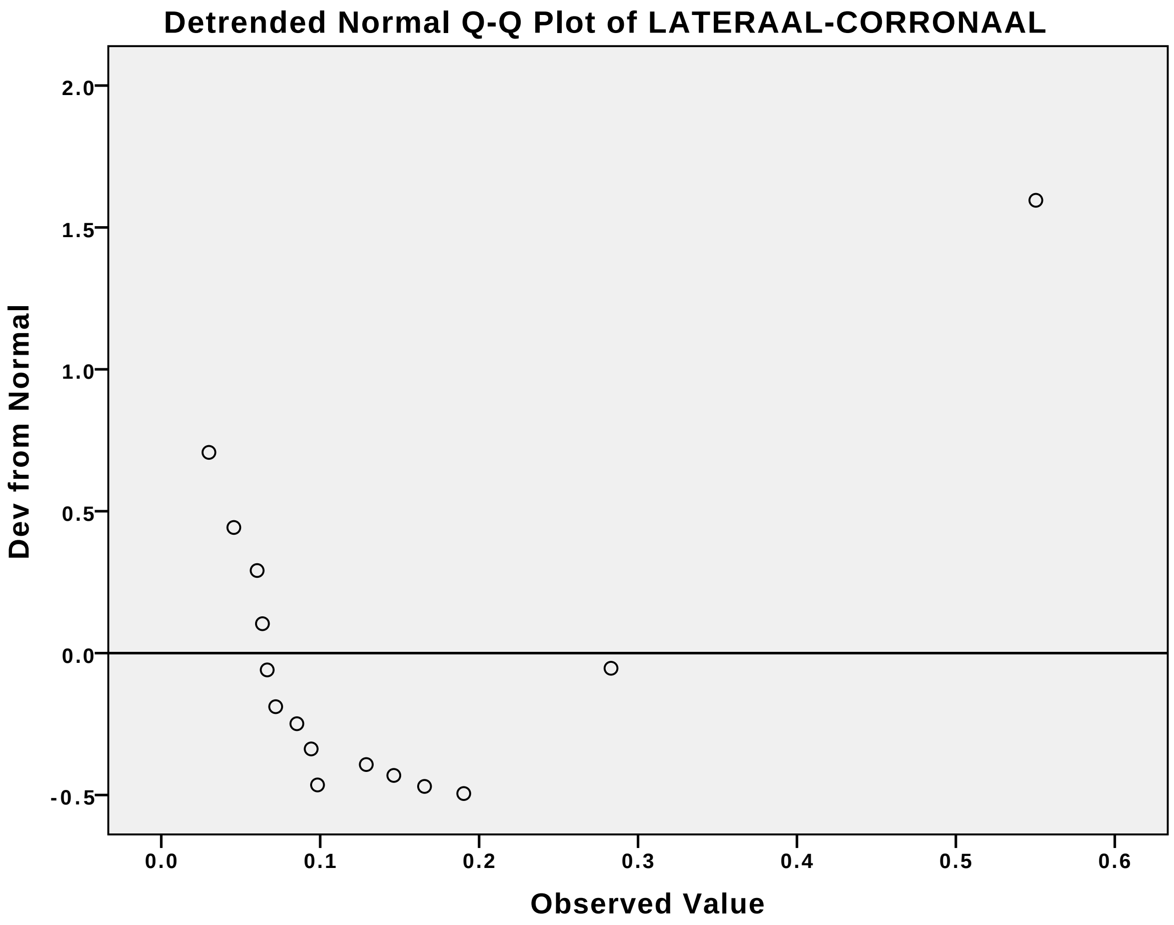
Detrended Normal Q-Q Plot of LATERAAL-CORRONAAL
Dev from Normal
2.0
1.5
1.0
0.5
0.0
-0.5
0.0
0.1
0.2
0.3
0.4
0.5
0.6
Observed Value

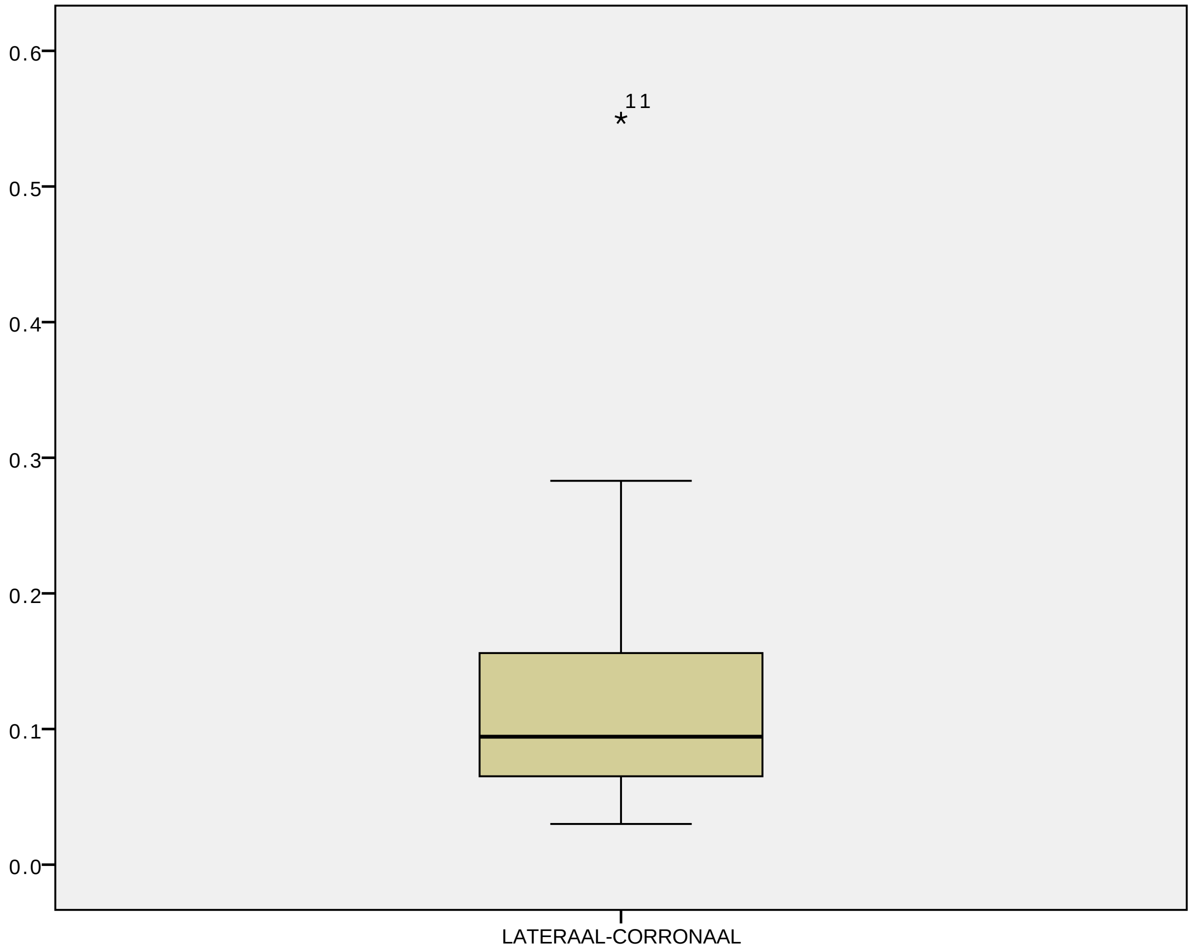


```
 NPAR TESTS
  /WILCOXON=LATERAALTRANSVERSAAL LENGTETRANSVERSAAL DIEPTESAGGITAAL WITH LA
TERAALCORRONAAL
    LENGTESAGGITAAL DIEPTECORONAAL (PAIRED)
  /MISSING ANALYSIS.
```

NPar Tests

Notes

Output Created		10-APR-2017 13:56:...
Comments		
Input	Active Dataset	DataSet7
	Filter	<none>
	Weight	<none>
	Split File	<none>
	N of Rows in Working Data File	18
Missing Value Handling	Definition of Missing	User-defined missing values are treated as missing.
	Cases Used	Statistics for each test are based on all cases with valid data for the variable(s) used in that test.
Syntax		NPAR TESTS /WILCOXON=LATERAAL TRANSVERSAAL LENGTETRANSVERSAAL DIEPTESAGGITAAL WITH LATERAALCORRONAAL LENGTESAGGITAAL DIEPTECORONAAL (PAIRED) /MISSING ANALYSIS.
Resources	Processor Time	00:00:00.01
	Elapsed Time	00:00:00.00
	Number of Cases Allowed[a]	285975

a. Based on availability of workspace memory.

Wilcoxon Signed Ranks Test

Ranks

		N	Mean Rank	Sum of Ranks
LATERAAL-CORRONAAL - LATERAAL - TRANSVERSAAL	Negative Ranks	11[a]	9.27	102.00
	Positive Ranks	7[b]	9.86	69.00
	Ties	0[c]		
	Total	18		
LENGTE - SAGGITAAL - LENGTE-TRANSVERSAAL	Negative Ranks	6[d]	6.67	40.00
	Positive Ranks	9[e]	8.89	80.00
	Ties	0[f]		
	Total	15		
DIEPTE-CORONAAL - DIEPTE - SAGGITAAL	Negative Ranks	6[g]	7.25	43.50
	Positive Ranks	12[h]	10.63	127.50
	Ties	0[i]		
	Total	18		

a. LATERAAL-CORRONAAL < LATERAAL - TRANSVERSAAL

b. LATERAAL-CORRONAAL > LATERAAL - TRANSVERSAAL

c. LATERAAL-CORRONAAL = LATERAAL - TRANSVERSAAL

d. LENGTE - SAGGITAAL < LENGTE-TRANSVERSAAL

e. LENGTE - SAGGITAAL > LENGTE-TRANSVERSAAL

f. LENGTE - SAGGITAAL = LENGTE-TRANSVERSAAL

g. DIEPTE-CORONAAL < DIEPTE - SAGGITAAL

h. DIEPTE-CORONAAL > DIEPTE - SAGGITAAL

i. DIEPTE-CORONAAL = DIEPTE - SAGGITAAL

Test Statistics[a]

	LATERAAL-CORRONAAL - LATERAAL - TRANSVERSAAL	LENGTE - SAGGITAAL - LENGTE-TRANSVERSAAL	DIEPTE-CORONAAL - DIEPTE - SAGGITAAL
Z	-.719[b]	-1.136[c]	-1.830[c]
Asymp. Sig. (2-tailed)	.472	.256	.067

a. Wilcoxon Signed Ranks Test

b. Based on positive ranks.

c. Based on negative ranks.
